# Supplementary material for: The inducible amphisome isolates viral hemagglutinin and defends against influenza A virus infection
Source: Nat Commun. 2020 Jan 9;11:162. doi: 10.1038/s41467-019-13974-w (PMC6952414; doi:10.1038/s41467-019-13974-w)
Supplement: Supplementary file 1 — Supplementary Information [file 41467_2019_13974_MOESM1_ESM.pdf]

The Inducible Amphisome Isolates Viral Hemagglutinin and Defends Against Influenza A Virus Infection

J. Omi et al.

Supplementary Information

・Supplementary Table 1
・Supplementary Figures 1-18
・Supplementary References

Supplementary table 1. Primers and oligo DNAs used in this study.

Primer set for HA subcloning into pBacPAK8
Forward: AGAGGAATTCAACAAATTAGAAAAAAGGATGGAAA<br>Reverse: AGAGCCCGGGTCAGTGGTGGTGGTGGTGGATGCATATTCTGCACTGCAAAGAT
Primer set for HA subcloning into pcDNA3.1(-)
Forward: CCACCACACTGGACTAGTGGGATCCGCGAAAGCAG<br>Reverse: CCTTGCTCACCTTATCGTCGTCATCCTTGTAATCA
Primer set for mVenus subcloning into pcDNA3.1(-)
Forward: CGACGATAAGGTGAGCAAGGGCGAGGAG<br>Reverse: AGCTTGGTACCGAGCTCGTCACTTGTACAGCTCGT
Primer set for canine *ABCA3* cDNA cloning
Forward1: CACCACACTGGACTAGTGTGGCCAGTCCTGCCATA<br>Forward2: ACACAGGTTCGAAGGCCTATTTGCCAAGCTGGAGA<br>Reverse1: ATAGGCCTTCGAACCTGTGTGTGCTCTC<br>Reverse2: CCTTGTA ATCCGGCCCCTCGTCCTCGGC
OligoDNAs for constructing sg*ULK1* expression vector
sg*ULK1*: CACCGCGGAATTGGCCATTTCCTGC<br>sg*ULK1*-Complementary: AAACGCAGGAAATGGCCAATTCCGC
OligoDNAs for constructing sg*PIK3C3* expression vector_1
sg*PIK3C3*: CACCGGGCAGGTCAGGGTACTTTAC<br>sg*PIK3C3*-Complementary: AAACGTAAAGTACCCTGACCTGCCC
OligoDNAs for constructing sg*PIK3C3* expression vector_2
sg*PIK3C3*: CACCGCCCAGGTGGCCCTCACTATA<br>sg*PIK3C3*-Complementary: AAACTATAGTGAGGGCCACCTGGGC
Primer set for qPCR of canine *ABCA3*
Forward: AAGGAGTACATGCGCATGA<br>Reverse: TGACCATGAAGCTGAAGGA
Oligo DNAs for the construction of *ABCA3*-targeting sgRNA expression
sg*ABCA3*: CACCGCGAGGGAAAGCCTCGAGCTG<br>sg*ABCA3*-Complementary: AAACCAGCTCGAGGCTTTCCCTCGC
Sequencing primer for *ABCA3* cDNA
AGAGGAATTCCGGCGCGGAGGACATTGGTC
Primers set for genomic PCR or sequencing of *Abca3* genome
Forward: TGTCCTTGCGGCAGCCAATC<br>Reverse: TGGTTTCCTGGCTTCATGCTG
Primer set for qPCR of IAV *NP*
Forward: GACGATGCAACGGCTGGTCTG<br>Reverse: ACCATTGTTCCAACTCCTTT
OligoDNAs for constructing sg*ABCA3* expression vector
Forward: CACCGCGAGGGAAAGCCTCGAGCTG<br>Reverse: AAACCAGCTCGAGGCTTTCCCTCGC

Supplementary Figure 1

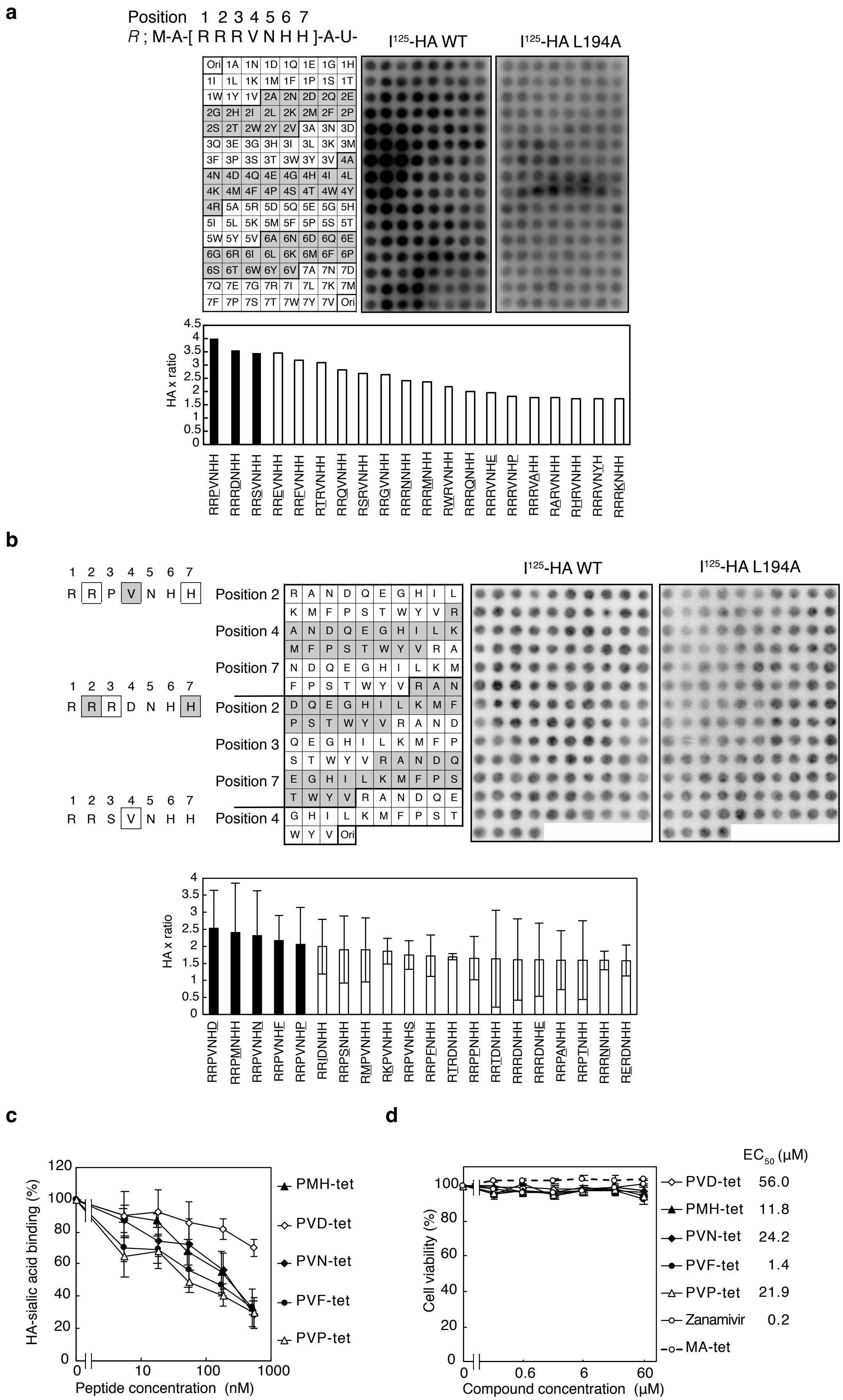

Supplementary Figure 1. Identification of high-affinity HA-binding motifs by screening tetravalent peptides synthesized on a cellulose membrane.

a For the first screen, each amino acid present in the HA-binding motif of RVH-tet, RRRVNHH, was replaced individually by all amino acids except Cys. The position and the substituted amino acid are shown in the upper left panel. Ori indicates the original motif. The tetravalent peptides with the indicated sequences were synthesized on the corresponding position of a membrane (upper middle and right panels). The membrane was blotted with ^{125}I-labeled HA (^{125}I-HA) or ^{125}I-labeled HA L194A (^{125}I-HA L194A) (1 μg/ml), and the radioactivity of each bound peptide spot was quantified as a pixel value using a BAS-2500 bio-imaging analyzer (GE Healthcare Sciences, USA). The sum of the pixel values of all the peptide spots was normalized to 127 (the number of tetravalent peptides synthesized on the membrane) so that each peptide would have a value of 1 in the absence of selectivity for HA. The ratio of ^{125}I-HA binding to ^{125}I-HA L194A binding was calculated, and the sum of each ratio was normalized to 127 to evaluate the specificity of binding through L194. The product of the HA-binding value and the normalized ratio (HA × ratio) was used to evaluate both the binding intensity and the specificity. The sequences were sorted in descending order based on the HA × ratio values. The HA × ratio values of the top 20 sequences are shown (lower panel). All of the top 15 sequences have an amino acid substitution at position 2, 3, 4, or 7. Based on that observation, the second library was designed as follows.

b For the second screen, each amino acid at positions 2, 4, and 7 of RRPVNHH, positions 2, 3, and 7 of RRRDNHH, and position 4 of RRSVNHH was replaced individually with all amino acids except Cys (upper left panel). Ori indicates the original motif. The tetravalent peptides with the indicated sequences were synthesized at the corresponding position of a membrane (upper middle and right panels). The membrane was blotted with ^{125}I-HA or ^{125}I-HA L194A and analyzed as described above. The HA × ratio values of the top 20 sequences are shown (mean ± SEM of three independent experiments, lower panel).

c AlphaScreen assay to examine the inhibitory effect of tetravalent peptides on the binding between HA and its receptor mimic, α2-6sialyllactose-PAA. Data are presented as a percentage of the control value (mean ± SEM of three independent experiments).

d The cytotoxicity of the compounds was assessed by incubating MDCK cells with the indicated amounts of each compound for 24 h. Data are presented as a percentage of the control value without compound (mean ± SEM of four independent experiments). Source data are provided as a Source Data File.

Supplementary Figure 2

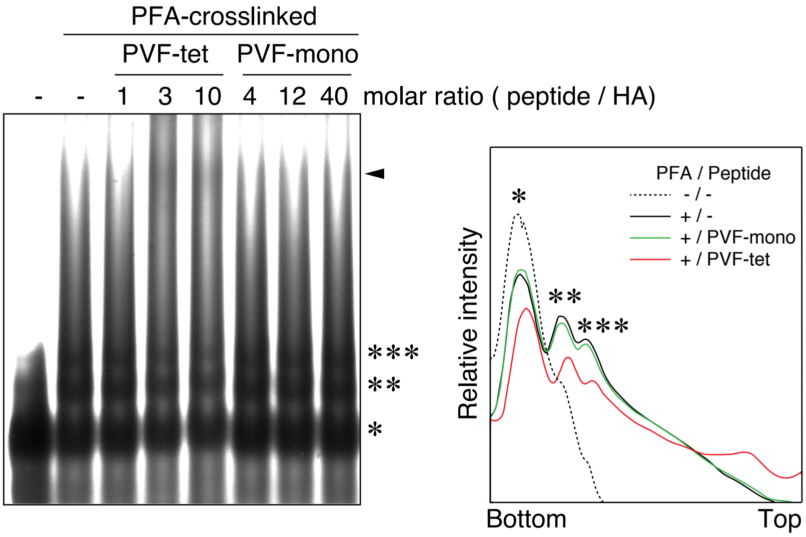

Supplementary Figure 2. PVF-tet induces the formation of highly clustered HA.

HA (2 μM) was incubated with the indicated amounts of PVF-tet or PVF-mono at 4°C for 1 h. The reaction mixtures were fixed with 1% PFA to avoid dissociation during subsequent SDS-PAGE analysis, and then denatured by mixing with Laemmli sample buffer for 10 min at room temperature. The mixtures were separated using 2% polyacrylamide gel supported with 1% agarose[1], and then detected by silver staining (left panel). The densitometric intensity of each lane was analyzed using ImageJ software (NIH, USA) (right panel). The asterisks indicate HA monomer (*), dimer (**), and trimer (***). The high-molecular-weight smeared band (arrowhead), which represents highly clustered HA, was observed only in the presence of PVF-tet at levels that were dose dependent.

Supplementary Figure 3

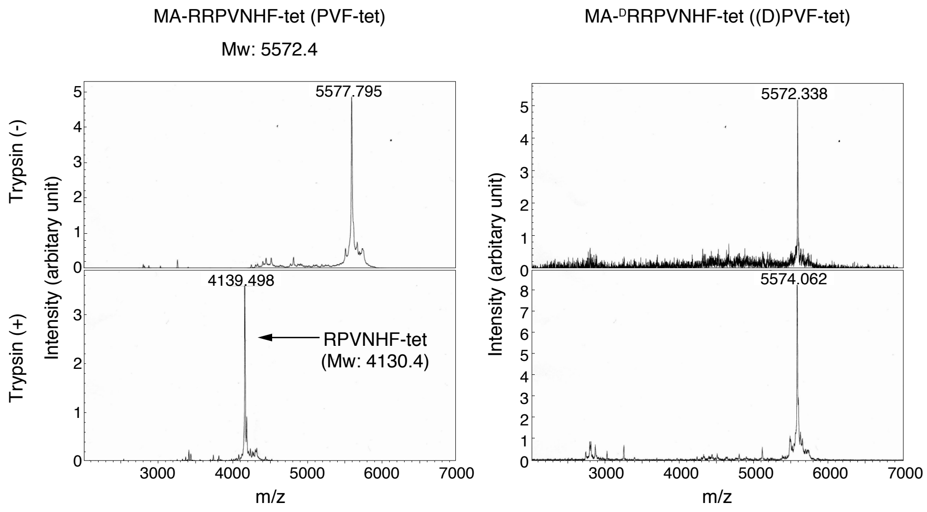

Supplementary Figure 3. Substitution of the first Arg of the RRPVNHF motif with D-Arg resulted in complete resistance to trypsin.

PVF-tet or (D)PVF-tet (20 μM) was treated with 1 μg/ml trypsin in Hank's balanced salt solution at 37°C for 24 h. After the treatment, mass spectrometric analysis was performed using the autoflexII TOF/TOF system (Bruker Daltonics).

Supplementary Figure 4

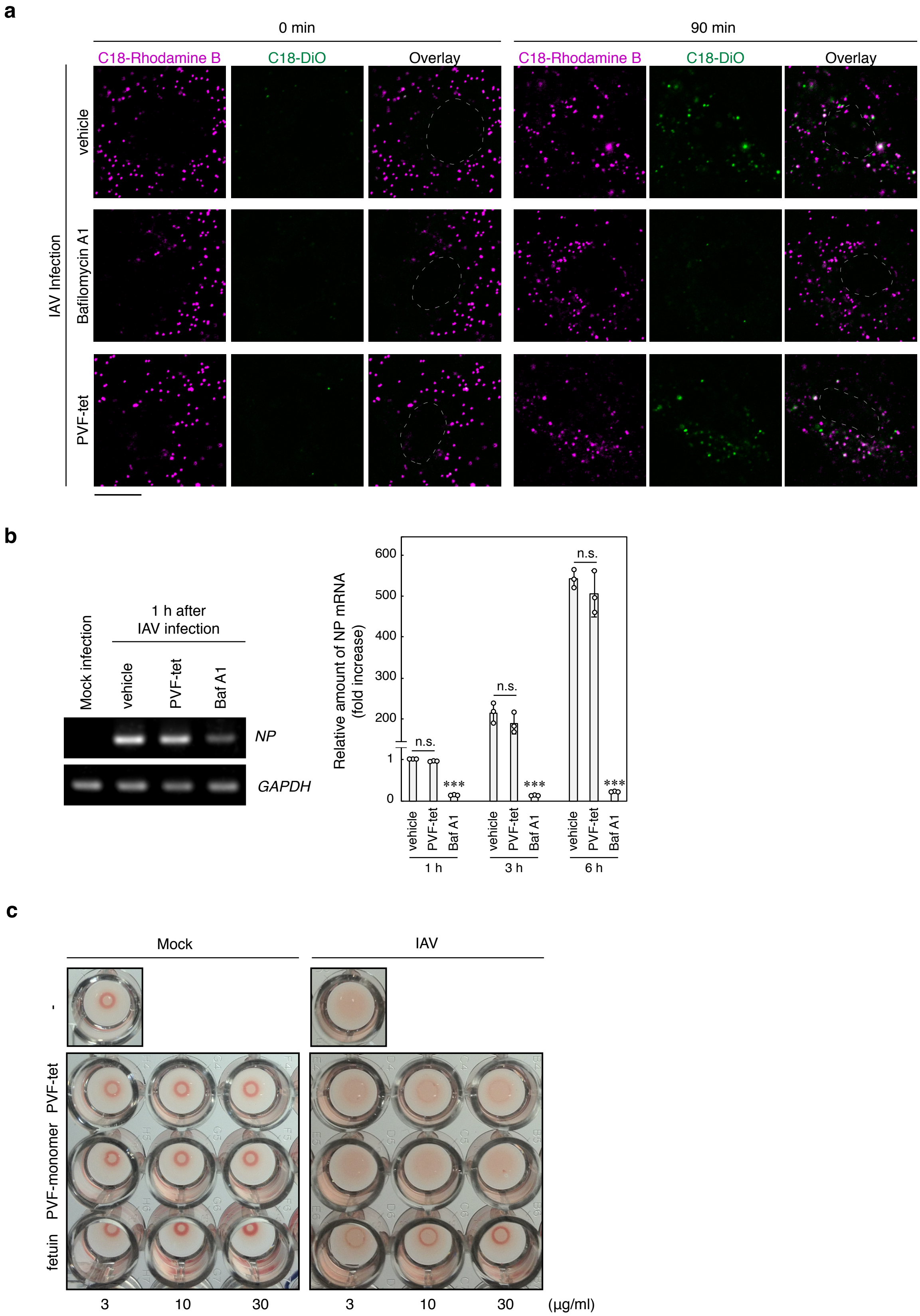

Supplementary Figure 4. PVF-tet does not affect the binding or entry of IAV.

a To determine the effects of PVF-tet on the fusion process after endocytosis of the virus, IAV strain PR8 was propagated in the presence of trypsin (1 µg/ml) for 72 h and then purified using chicken red blood cells (cRBC)[2]. Purified IAV particles (5 µg of total viral proteins) were labeled with two different lipophilic tracers, C18-Rhodamine B (red fluorescence) and C18-DiO (green fluorescence)[3]. The IAV can be detected as a particle with red fluorescence only, because the fluorescence of DiO is quenched by that of Rhodamine B through the Förster resonance energy transfer. MDCK cells were infected with the labeled IAV at 10 MOI in the presence of PVF-tet (20 µM), bafilomycin A1 (100 nM), or vehicle for 30 min at 4°C. After washing (0 min), the cells were cultured at 37°C in the presence of PVF-tet (20 µM), bafilomycin A1 (100 nM), or vehicle for 90 min. Fluorescent images were analyzed using laser scanning confocal microscopy. After fusion of the virus with the endosome membrane, these two tracers diffuse into the endosomal membrane, allowing the endosome to be detected by both red and green fluorescence as a result of DiO dequenching. Dashed lines indicate the nucleus. Scale bar represents 10 µm.

b Quantification of NP mRNA level. MDCK cells were infected with IAV strain PR8 at 10 MOI in the presence of PVF-tet (20 µM), bafilomycin A1 (100 nM), or vehicle for 1 h at 4°C. After washing, the cells were cultured at 37°C in the presence of the same compounds. After the indicated time periods, the cells were lysed in Sepasol RNAI Super G (Nacalai tesque, Japan). Agarose gel electrophoresis was performed for the detection of virus *NP* (left panel), and RT-qPCR (right panel) was performed using specific primers (Supplementary table. 1)[4]. Data were analyzed by relative quantification based on the ddCt methods using *GAPDH* as a reference gene, and expressed as a fold increase over the average mRNA level of the IAV infected cells recovered after 1 h incubation at 37°C without any compounds (mean ± SD of three independent experiments). ***$P < 0.001$ (compared with vehicle treatment by ANOVA followed by two-sided Dunnett's test). n.s., not significant.

c The effects of PVF-tet on IAV-induced hemagglutination. One HA unit of IAV particles (strain PR8) was pre-incubated with various concentrations of PVF-tet, PVF-monomer, or fetuin at 4°C for 1 h and then incubated with equal volumes of 0.75% chicken red blood cells at 4°C for 1 h in a U-bottom 96-well plate. Hemagglutination was then visually analyzed (right panel). The left panel shows mock (no virus) controls. One HA unit was defined as the minimum amount of IAV particles that caused hemagglutination.

Source data are provided as a Source Data File.

Supplementary Figure 5

a

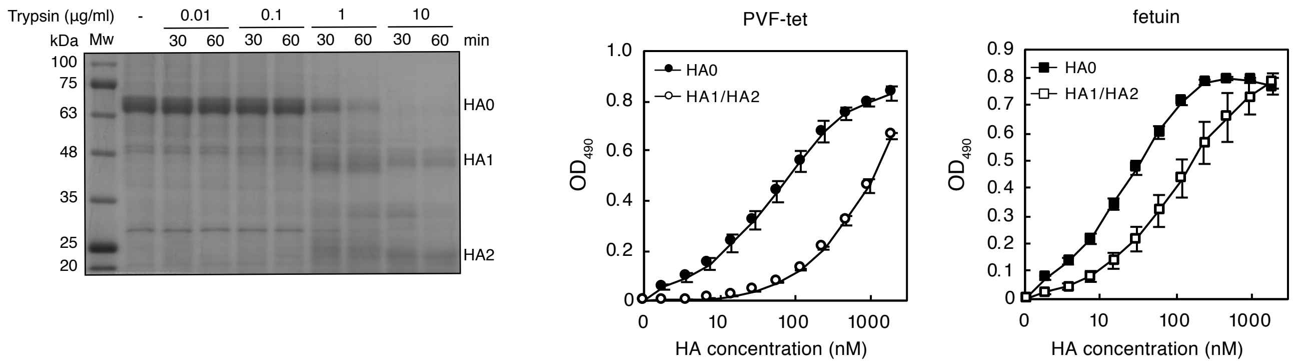

b

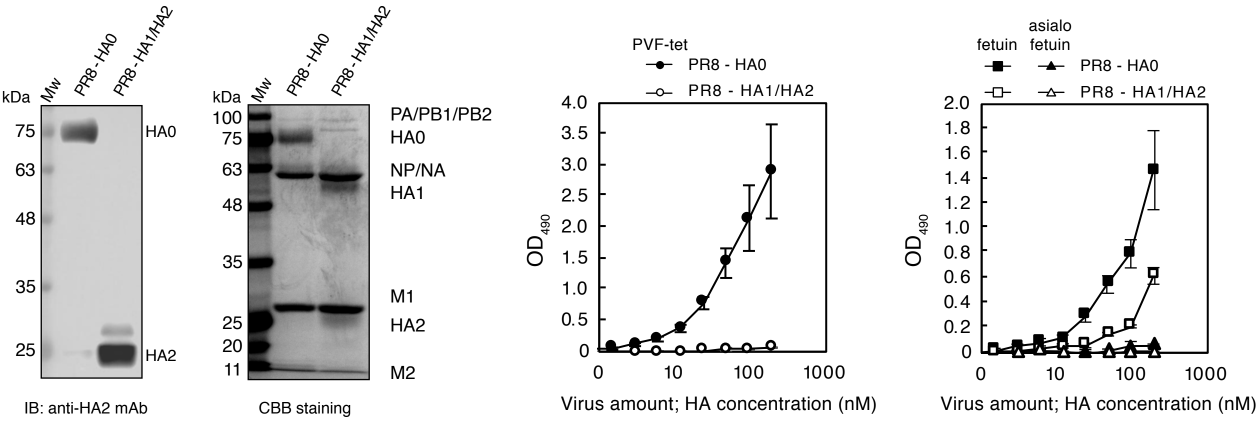

Supplementary Figure 5. The proteolytic cleavage of HA reduces HA binding to PVF-tet or sialic acid.

a (left panel) Preparation of trypsin-cleaved HA. Recombinant HA0 (6 μM) was treated with various concentrations of trypsin at 37°C for the indicated time periods. Treatment with 10 μg/ml trypsin for 30 min caused complete cleavage of HA0 into HA1/HA2. After the reaction was stopped with leupeptin (40 μM), HA1/HA2 was quantified by BCA assay and then used for ELISA. (right panels) ELISA to measure the binding of the indicated concentrations of HA0 or HA1/HA2 to PVF-tet (2 μM) or fetuin (0.3 μM) immobilized on a plate as described in the Methods. Bound HA was detected using anti-His-tag antibody (clone 9C11, Wako) followed by horseradish peroxidase (HRP)-conjugated horse anti-mouse IgG antibody. Data are shown as the mean ± SEM of three independent experiments. The binding of HA to PVF-tet was reduced by the cleavage of HA. The binding of HA to fetuin was also reduced by the cleavage of HA, although to a lesser degree than the binding to PVF-tet.

b (left panel) Preparation of biotinylated IAV strain PR8 with HA0 (PR8-HA0) or HA1/HA2 (PR8-HA1/HA2). PR8-HA0 was propagated in the presence of 10% FBS for 24 h and then purified using chicken red blood cells as described previously (Protocol Exchange, DOI: 10.1038/protex.2014.027). Biotinylated PR8-HA0 was prepared using biotin-$(AC5)_2$-Sulfo-OSu (Dojindo) according to the manufacturer's protocol. The biotinylated PR8-HA0 was then treated with 10 μg/ml trypsin for 30 min to produce PR8-HA1/HA2 without any loss of other viral proteins. Excess biotin-$(AC_5)_2$-Sulfo-Osu and trypsin were removed by ultracentrifugation. The amount of HA in the purified samples of biotinylated PR8-HA0 and PR8-HA1/HA2 was quantified by densitometry using ImageJ. (right panels) ELISA to measure the binding of biotinylated PR8-HA0 or PR8-HA1/HA2 to PVF-tet (2 μM), fetuin (0.3 μM), or asialofetuin (0.3 μM) immobilized on a plate. Bound IAV particles were detected using HRP-conjugated streptavidin (Dako). Data are presented as the mean ± SEM of three independent experiments. All procedures were performed in the presence of zanamivir (40 μM) at 4°C to inhibit neuraminidase activity. The binding of PR8-HA0 to fetuin was much higher than that of infectious PR8-HA1/HA2, whereas neither virus exhibited any binding to asialofetuin.

Source data are provided as a Source Data File.

Supplementary Figure 6

a

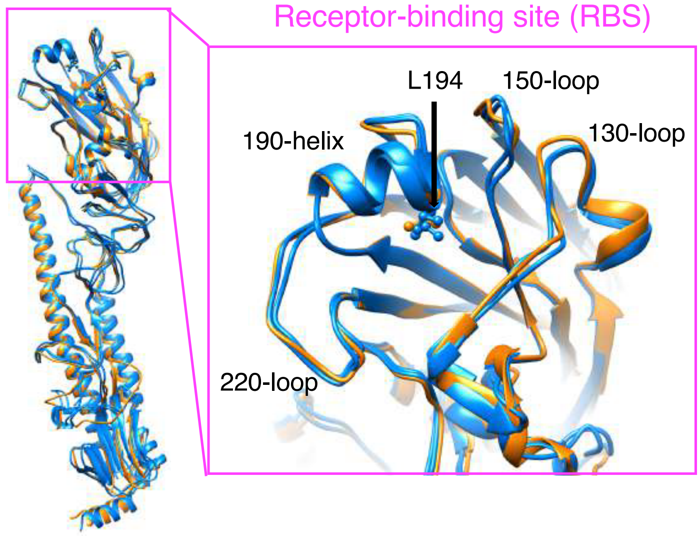

Subtype: H1	HA0	HA1/HA2	HA1/HA2
PBD ID	1RD8	1RU7	3LZG
Strain	A/Brevig Mission /1/1918	A/Puerto Rico/8/1934	A/California /1/1918
Space group	*C* 1 2 1	*C* 1 2 1	*P* 1
Resolution (Å)	3.0	2.3	2.6

b

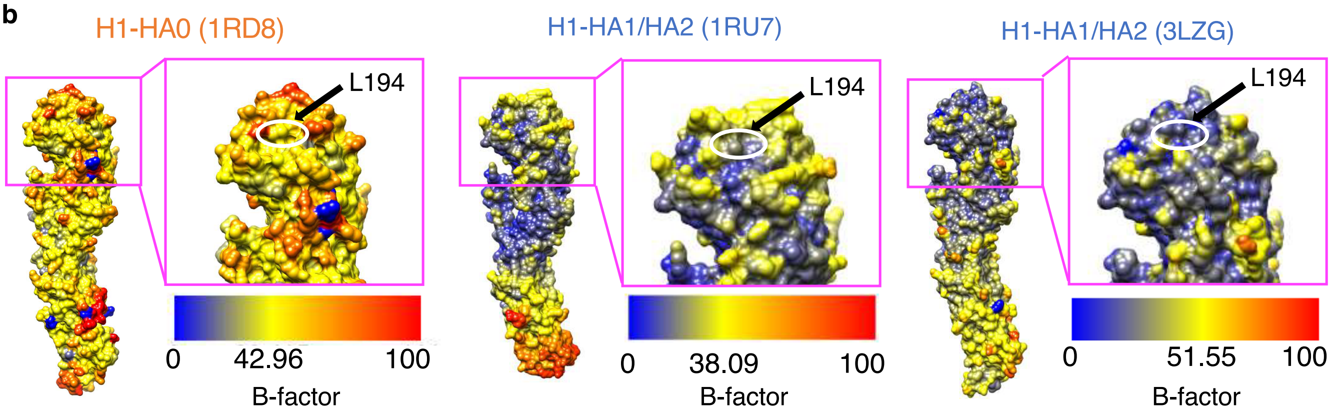

c

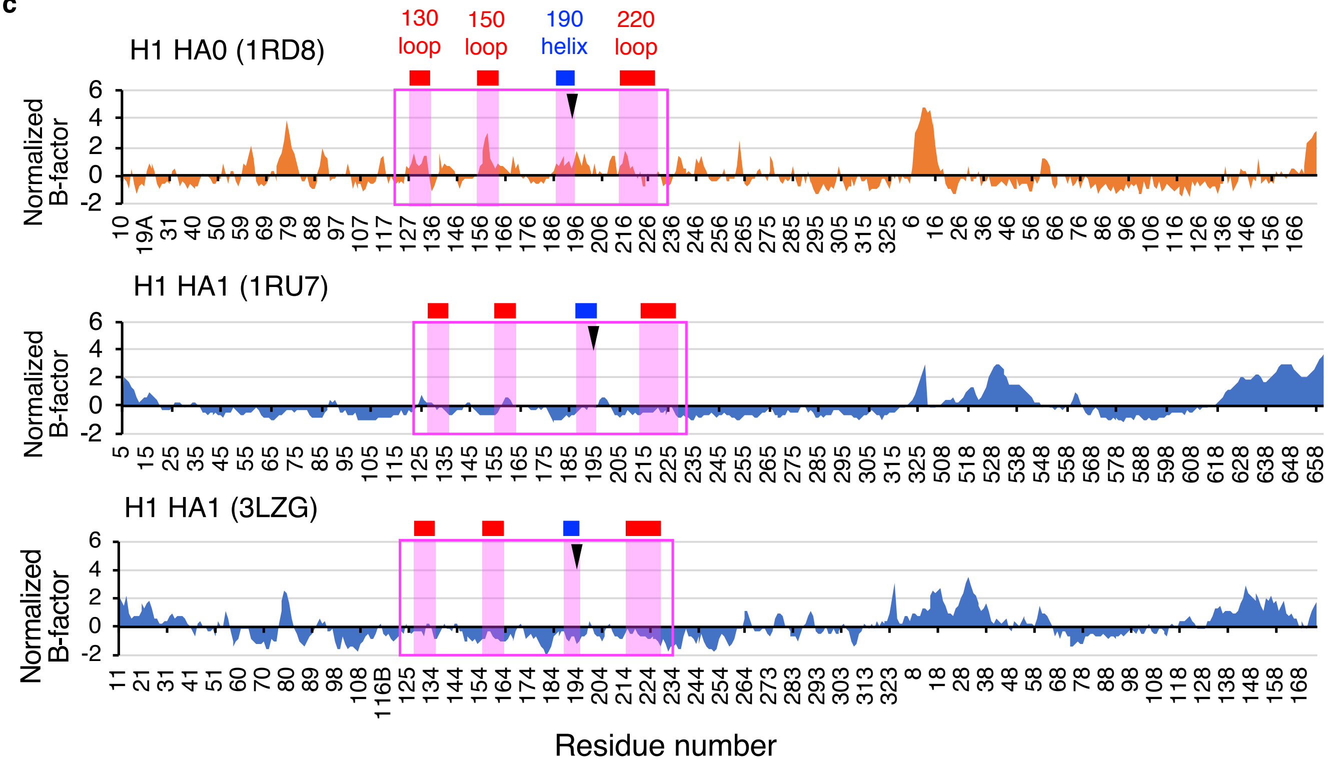

Supplementary Figure 6 continued

d

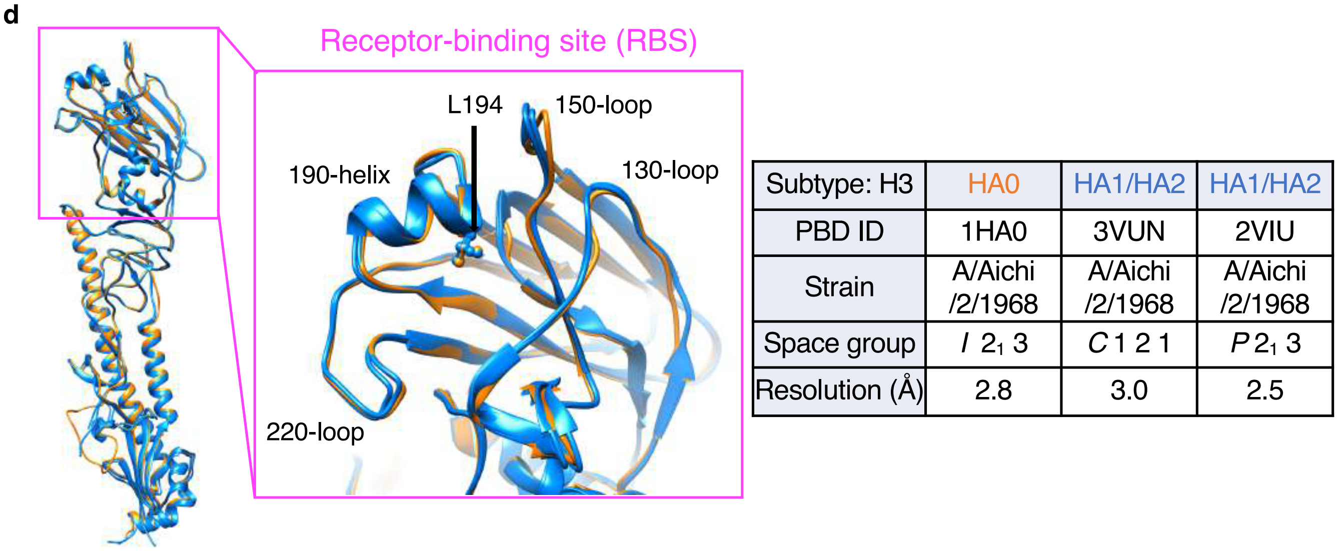

Subtype: H3	HA0	HA1/HA2	HA1/HA2
PBD ID	1HA0	3VUN	2VIU
Strain	A/Aichi /2/1968	A/Aichi /2/1968	A/Aichi /2/1968
Space group	$I\ 2_1\ 3$	$C\ 1\ 2\ 1$	$P\ 2_1\ 3$
Resolution (Å)	2.8	3.0	2.5

e

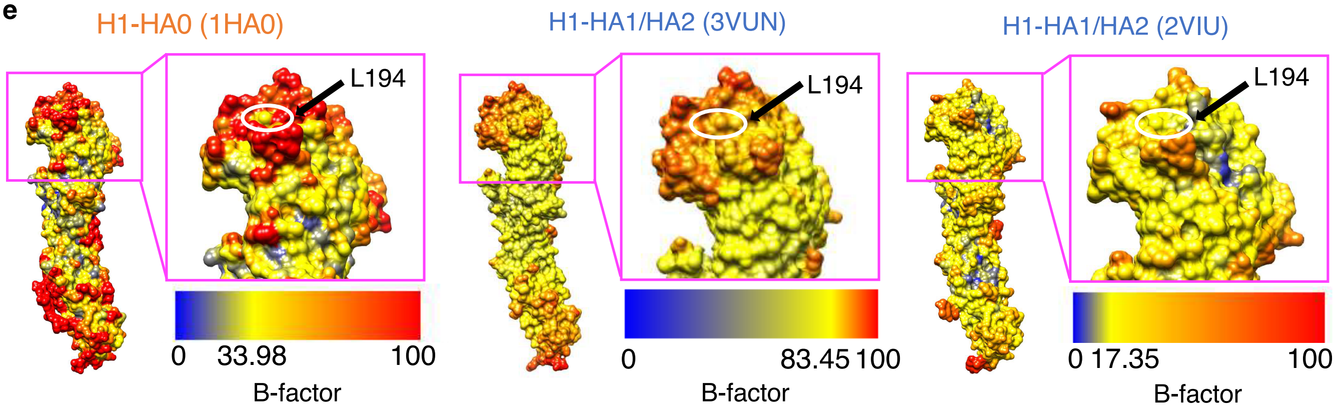

f

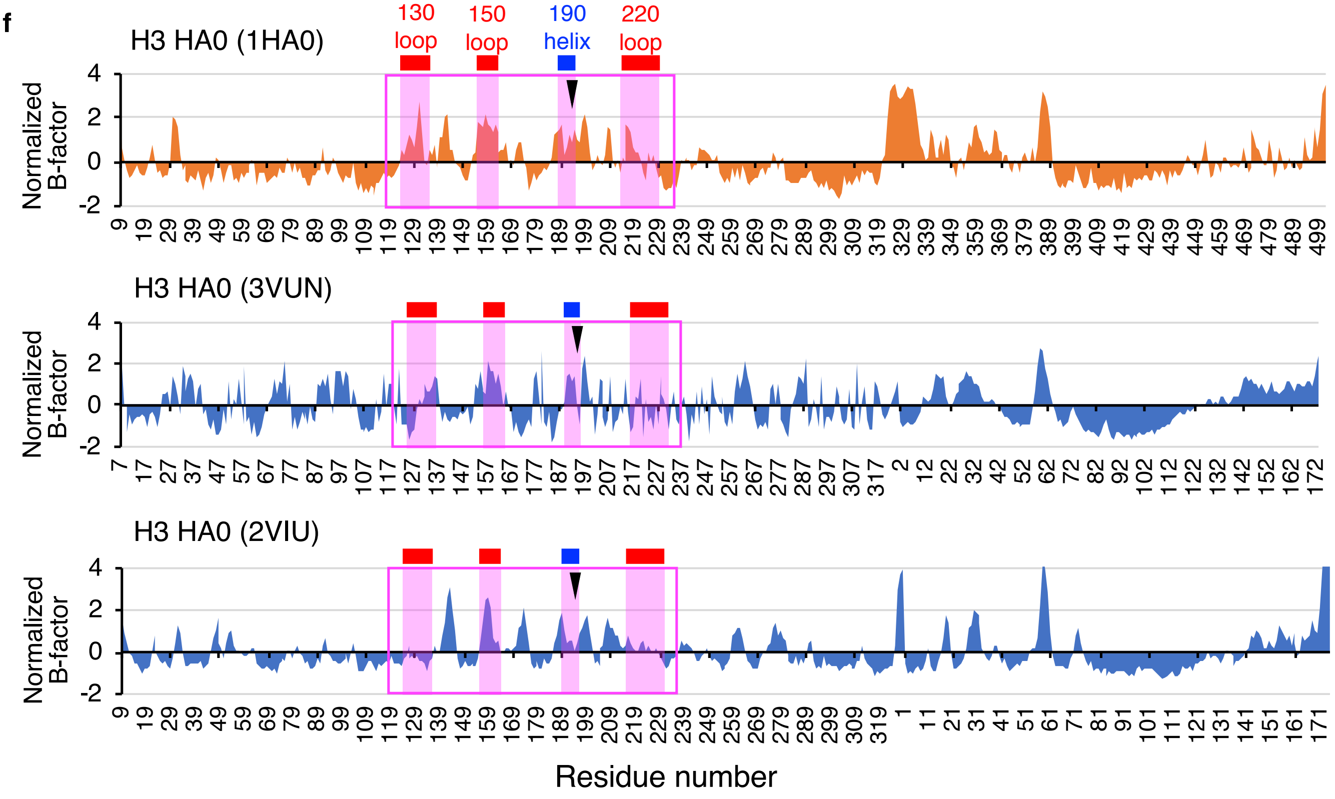

Supplementary Figure 6. Structural insights into HA0 and HA1/HA2.

a, d Comparison of the overall structures of HA0 and HA1/HA2 derived from (**a**) H1HA or (**d**) H3HA. **(a)** The crystal structures of H1HA from IAV H1N1 strain A/Brevig Mission/1/1918 (PDB: 1RD8) and (**d**) H3HA from IAV H3N2 strain A/Aichi/2/1968 (PDB: 1HA0) were used as the representative HA0 structures. **(a)** The crystal structures of H1HA from IAV H1N1 strains A/Puerto Rico/8/1934 (PDB: 1RU7) and A/California/04/2009 (PDB: 3LZG), along with (**d**) H3HA from IAV H3N2 strain A/Aichi/2/1968 (PDB: 3VUN and 2VIU), were used as the representative HA1/HA2 structures. In addition to H1HA from H1N1 strain A/Puerto Rico/8/1934, H1HA from H1N1strain A/California/04/2009 and H3HA from H3N2 strain A/Aichi/2/1968 are also shown to form inducible amphisomes in the presence of PVF-tet after infection as described in Supplementary Fig. 16. HA0 (orange) and HA1/HA2 (blue) structures were superimposed as shown in the left panel. The middle panel depicts a close-up view of the receptor binding site (RBS) (enclosed in a box in the left panel), which consists of a 130-loop, a 150-loop, a 190-helix including L194, and a 220-loop. The characteristics of each HA PDB entry are shown in the table at right. In both cases, the cleavage of HA does not affect the structure of HA-RBS.

b, e Atomic fluctuation of HA0 and HA1/HA2 derived from (**b**) H1HA or (**e**) H3HA. The atomic fluctuation was assigned in terms of B-factors. The average B-factor in each PDB entry (1RD8, 1RU7 and 3LZG in **b**, and 1HA0, 3VUN, and 2VIU in **e**) was calculated using the B-factors of Cα atoms in a single molecule. The structure of each individual HA is shown with a colored horizontal scale bar indicating the B-factors (the average B-factor is colored in yellow). The B-factors are categorized by HA subtype and PDB identifier in a Source Data File.

c, f Comparison of protein dynamics between sets of PDB entries for HA0 and HA1/HA2 derived from (**c**) H1HA or (**f**) H3HA. B-factor normalization was performed based on the conventional "z-score normalization" as follows: normalized B-factor = $[B_{x(i)} - \langle B \rangle_{(i)}] / s_{(i)}$, where $B_{x(i)}$ is the B-factor of atom x, $\langle B \rangle_{(i)}$ is the individual average B-factor calculated as described above, and $s_{(i)}$ is the standard deviation of the B-factors of Cα atoms in each structure (i)[5]. The normalized B-factors were plotted with the secondary structure of the RBS (circled with a magenta line), including the 130-loop, 150-loop, and 220-loop colored in red, and the 190-helix colored in blue. In both cases, the normalized B-factors in the HA0-RBS structure are higher than those in the HA1/HA2-RBS structure. The results imply that the higher atomic fluctuation of HA0-RBS compared with that of HA1/HA2-RBS leads to a more efficient interaction with sialic acid or PVF-tet (Supplementary Fig. 5) even though the crystal structures of HA0-RBS and HA1/HA2-RBS are

the same. HA cleavage changes the RBS to the less-fluctuated conformation, which strongly limits PVF-tet binding while exerting a weaker effect on sialic acid binding (Supplementary Fig. 5).

All structural figures were generated using the Chimera program[6].

Supplementary Figure 7

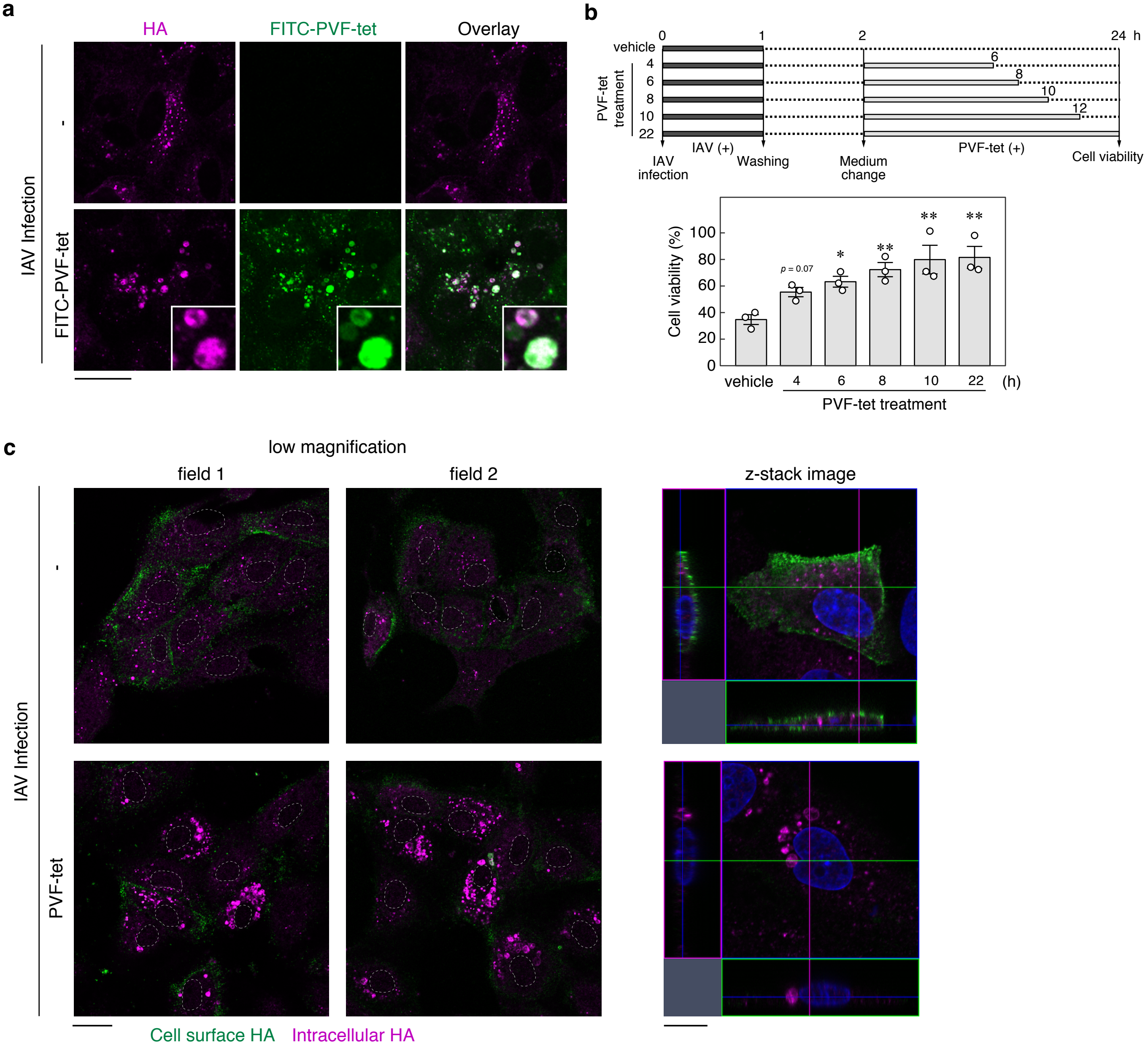

Supplementary Figure 7. PVF-tet induces the accumulation of newly synthesized HA in a vacuole-like structure.

a MDCK cells were infected with IAV strain PR8 at 10 MOI for 1 h at 37°C. After washing, the cells were cultured for 1 h. The cells were further cultured in the presence or absence of FITC-labeled PVF-tet (2.5 μM) for 4 h. After washing, the cells were cultured for 4 h and co-localization of HA with FITC-labeled (D)PVF-tet was analyzed by immunocytochemistry. Scale bar represents 20 μm.

b MDCK cells were infected with IAV strain PR8 at 10 MOI for 1 h at 37°C. After washing, the cells were cultured for 1 h. The cells were further cultured in the presence or absence of PVF-tet (20 μM) for the indicated time periods. After washing, the cells were cultured, and then the relative cell viability was determined at 24 h after infection. Detailed experimental setups are shown in the upper panel. Data are presented as a percentage of the control value (mean ± SEM of three independent experiments). *P < 0.05; **P < 0.01 (compared with vehicle treatment by ANOVA followed by one-sided Dunnett's test).

c Detection of cell surface HA and intracellular HA. To separately detect cell surface HA and intracellular HA, we used two different types of anti-HA antibody. MDCK cells were infected with IAV strain PR8 at 10 MOI for 1 h at 37°C. After washing, the cells were cultured in the presence or absence of PVF-tet (20 μM) for 15 h. The cells were treated with mouse anti-HA antibody (C102), followed by fixation with 3.75% PFA at room temperature for 20 min. After washing, the cells were treated with 0.3% TritonX-100, which can remove the cell surface HA but not anti-HA antibody (C102). Intracellular HA was sequentially stained with rabbit anti-HA antibody. Alexa488-labeled goat anti-mouse IgG (green) or Alexa546-labeled goat anti-rabbit IgG (red) was used as a secondary antibody for immunocytochemistry. Fluorescent images were analyzed using laser scanning confocal microscopy. The representative z-stack images were analyzed by scanning laser confocal microscopy (right panel); in each panel, left and lower images show cross-sectional images. Scale bars represent 20 μm (left) or 10 μm (right).

Source data are provided as a Source Data File.

Supplementary Figure 8

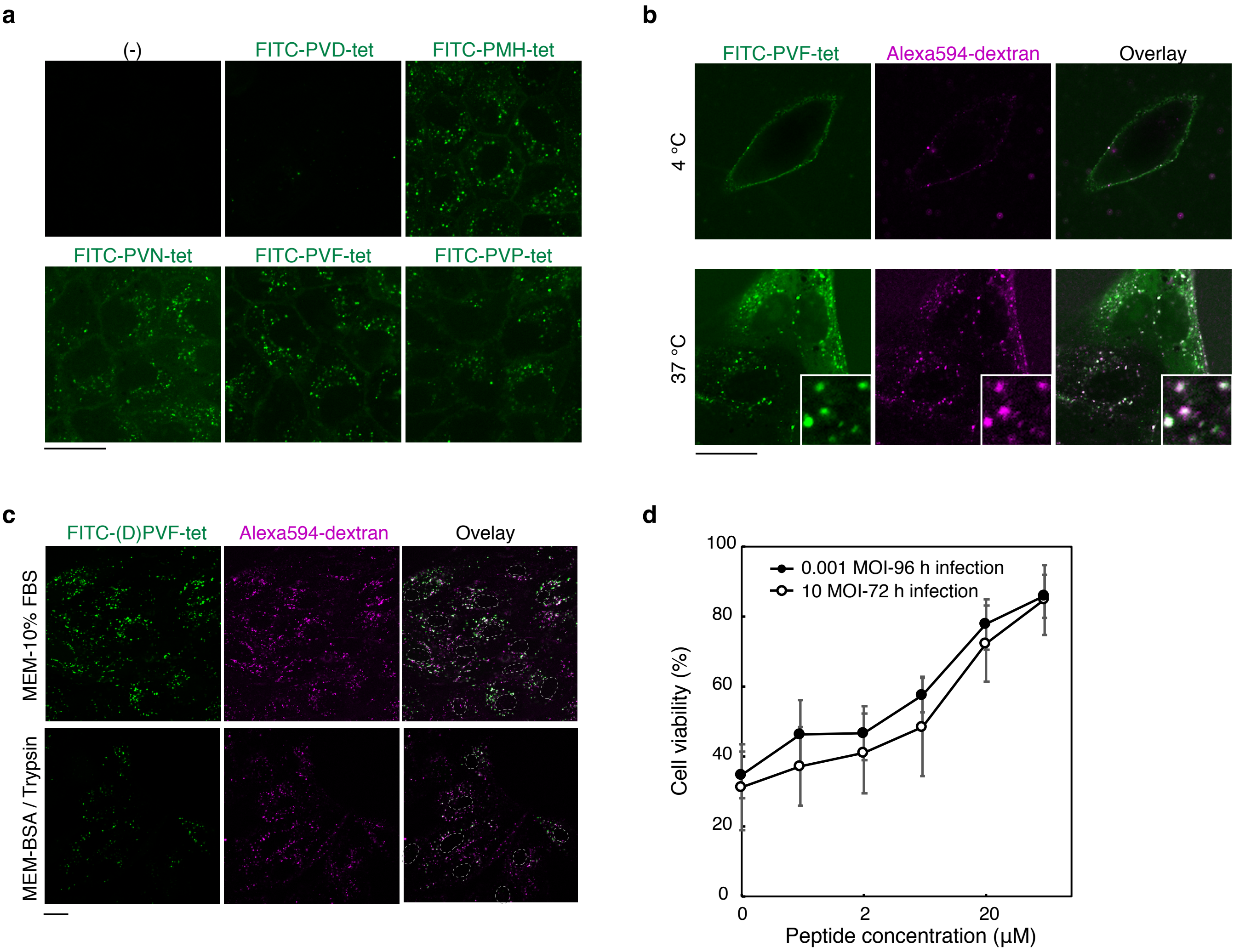

Supplementary Figure 8. PVF-tet penetrates into cells via the endocytic pathway.

a MDCK cells were incubated with each FITC-labeled peptide (2.5 μM) for 1 h at 37°C. Fluorescent images were obtained using confocal laser scanning microscopy to assess incorporation of tetravalent peptides into cells.

b MDCK cells were incubated with FITC-labeled PVF-tet (2.5 μM) in the presence of Alexa594-labeled dextran (250 μg/ml), which is widely known to be incorporated into cells via macropinocytosis, for 1 h at 4°C (upper panel) or 37°C (lower panel). Fluorescent images were obtained using laser scanning confocal microscopy to show co-localization. Insets show magnified fields.

c The effect of FBS on the incorporation of (D)PVF-tet. MDCK cells were incubated with FITC-labeled (D)PVF-tet (2.5 μM) and Alexa594-labeled dextran (250 μg/ml) in MEM supplemented with 10% heat-inactivated FBS, 50 units/ml penicillin, and 50 μg/ml streptomycin (Trypsin(-)-MEM), or serum-free MEM supplemented with 1 μg/ml trypsin, 0.2% BSA, 25 mM HEPES, 50 units/ml penicillin, and 50 μg/ml streptomycin (Trypsin(+)-MEM) for 1 h at 37°C. Fluorescent images were obtained using laser scanning confocal microscopy. Dashed lines indicate the nucleus.

d The effects of PVF-tet on the cytopathicity induced by IAV infection in Caco-2 cells. Caco-2 cells , which were cultured in DMEM supplemented with 10% FBS, 100 units/ml penicillin, 100 μg/ml streptomycin, and 25 μg/ml Fungizone, were incubated with the indicated concentrations of PVF-tet for 30 min, and then infected with IAV strain PR8 at 0.001 MOI for 96 h or 10 MOI for 72 h (right panel). After washing, the relative cell viability was determined. Data are presented as a percentage of the control value without infection (mean ± SEM of three independent experiments).

Scale bars represent 20 μm. Source data are provided as a Source Data File.

Supplementary Figure 9

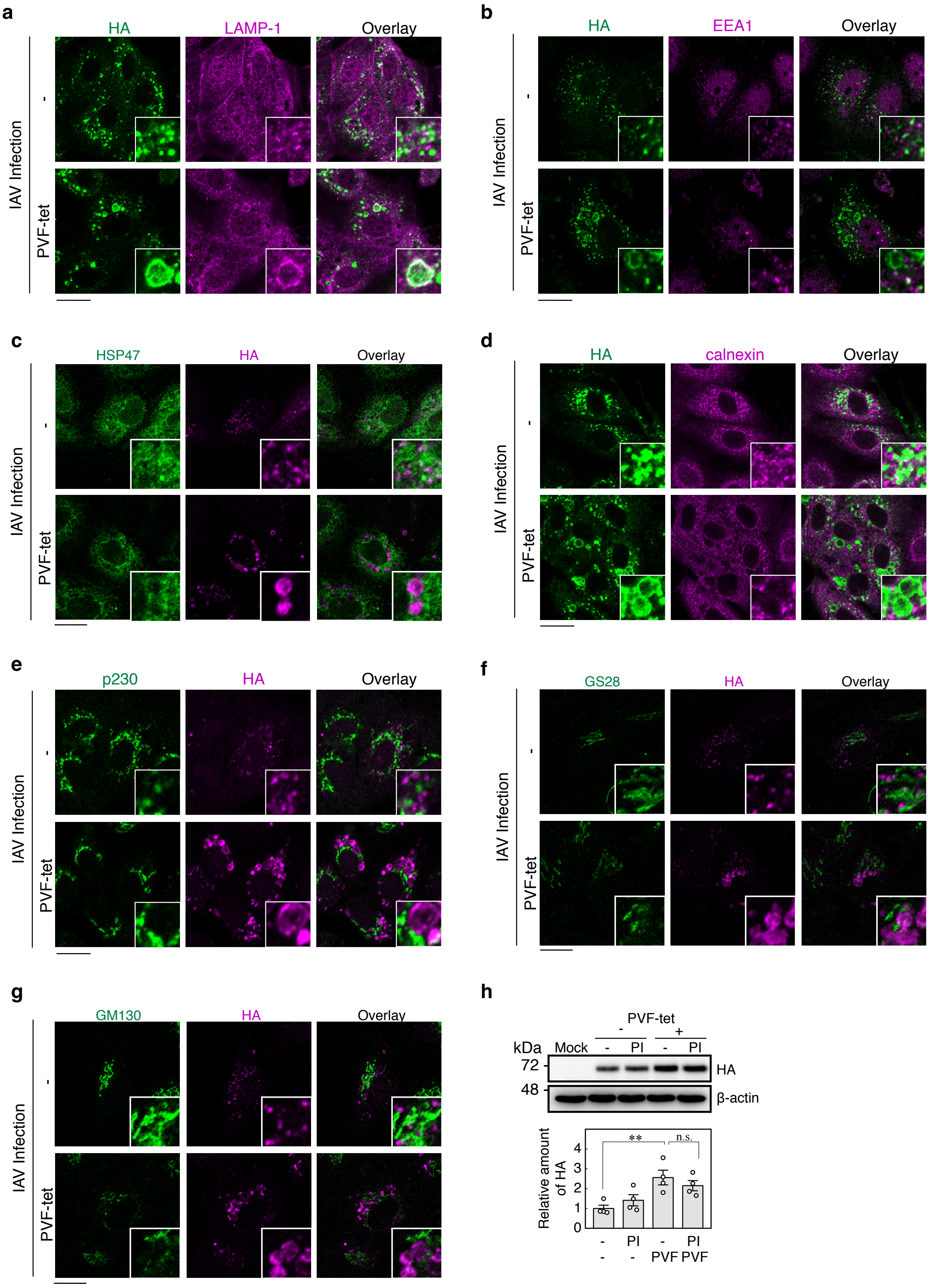

Supplementary Figure 9. PVF-tet-induced structure is a lysosome-related compartment but does not degrade HA.

a-g MDCK cells were infected with IAV strain PR8 at 10 MOI for 1 h. After washing, the cells were cultured in the presence or absence of PVF-tet (20 μM) for 15 h. The intracellular localization of HA was analyzed by immunocytochemical staining. Co-localization of HA with various organelle markers was analyzed by immunocytochemical staining using specific antibodies against markers such as (**a**) LAMP-1 (a lysosome marker; Abcam), (**b**) EEA1 (an early endosome marker; Affinity Bioreagents), (**c**) HSP47 (an ER marker; Enzo Lifesciences), (**d**) calnexin (an ER marker; Santa Cruz), (**e**) p230 (a *trans*-Golgi network marker; BD transduction), (**f**) GS28 (a *trans*-Golgi network marker; BD transduction), and (**g**) GM130 (a *cis*-Golgi network marker; BD transduction). Insets show magnified fields.

h MDCK cells were infected with IAV strain PR8 at 10 MOI for 1 h. After washing, the cells were cultured in the presence or absence of PVF-tet (20 μM) for 2 h. The cells were treated with or without lysosomal protease inhibitor (PI) cocktail containing 30 μM E-64, 15 μM Pepstatin A, and 20 μM Leupeptin and further cultured for 13 h. The cell lysates were analyzed by western blotting to measure the amount of cellular HA using a specific antibody (upper panel). The intensity of each band was quantified and data are presented as the amount of HA relative to that of β-actin (mean ± SEM of four independent experiments). **P < 0.01; *n.s.*, not significant (by one-way ANOVA followed by Tukey's test).

Scale bars represent 20 μm. Source data are provided as a Source Data File.

Supplementary Figure 10

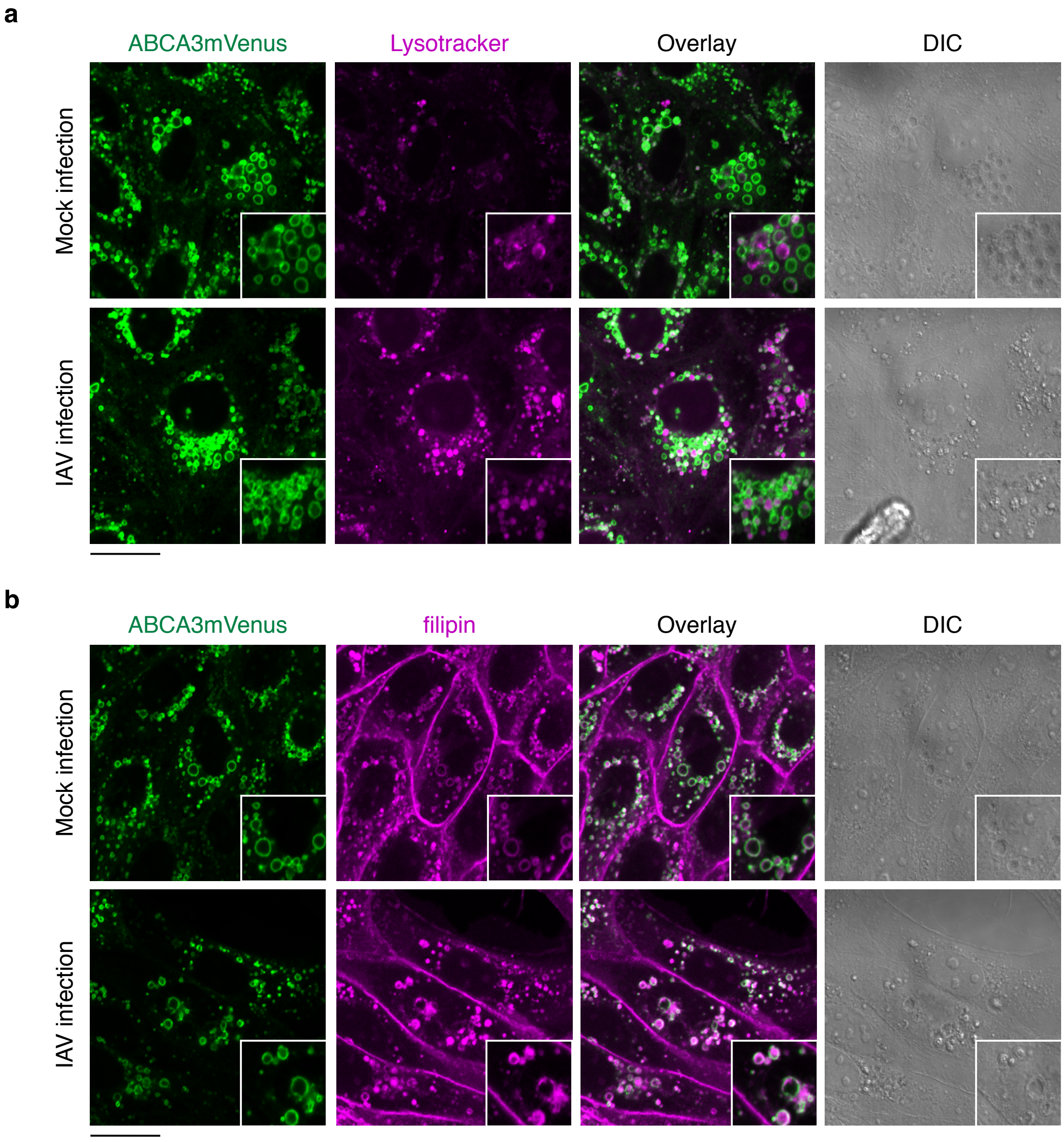

Supplementary Figure 10. The ABCA3-positive structure induced by IAV infection is highly acidified and enriched with lipids.

a Acidification of the ABCA3-positive structure was analyzed using Lysotracker.

b Co-localization of ABCA3mVenus with cholesterol was examined using filipin. Clone 6 stably overexpressing ABCA3mVenus was infected with IAV strain PR8 at 10 MOI for 1 h (lower panels) or not infected (upper panels). After washing, the cells were cultured for 15 h. Fluorescent images were analyzed using laser scanning confocal microscopy. Insets show magnified fields.

Scale bars represent 20 μm.

Supplementary Figure 11

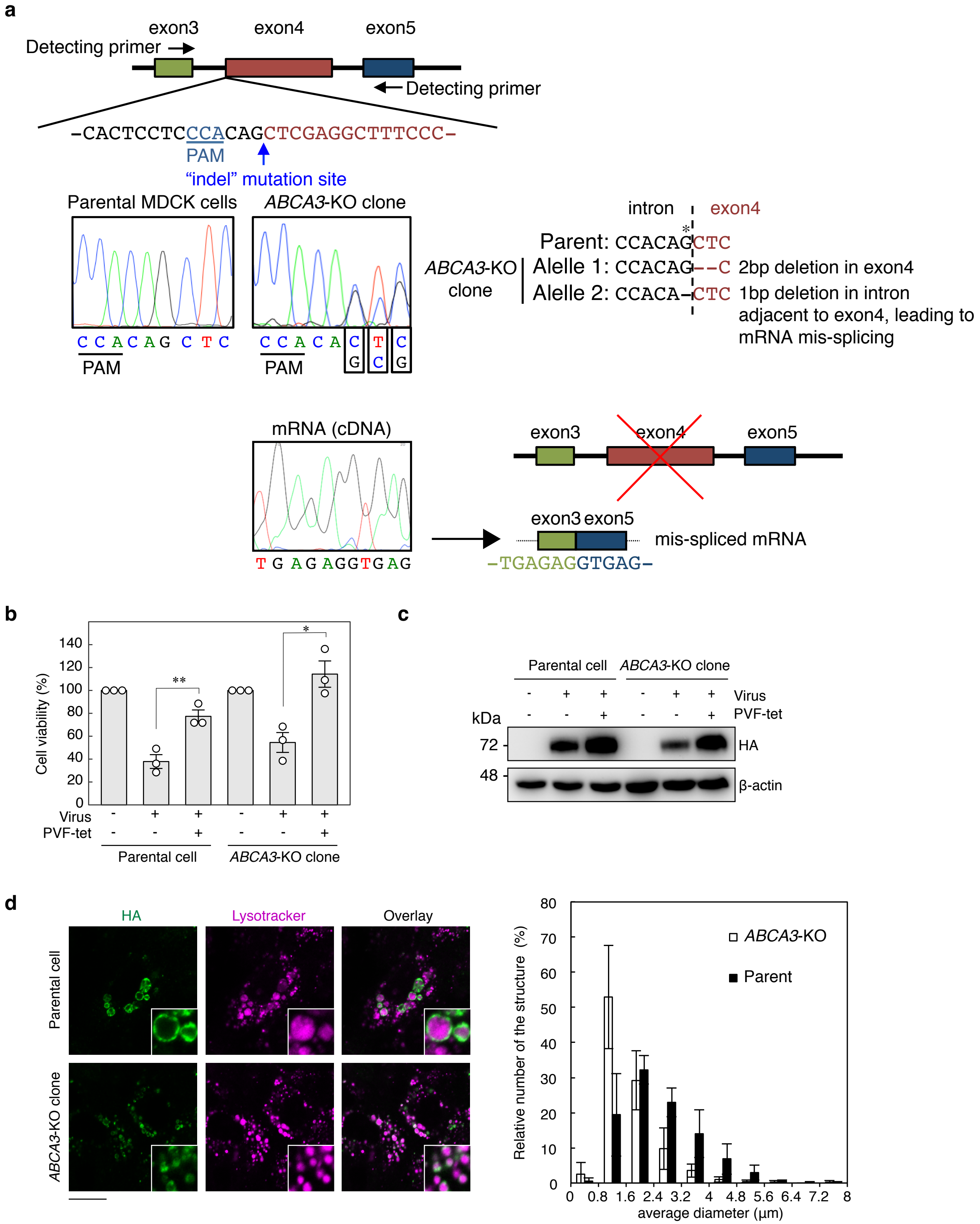

Supplementary Figure 11. Gene knockout of *ABCA3* does not affect the anti-IAV activity of PVF-tet, but reduces the average size of the HA-containing structure.

a CRISPR-Cas9-mediated genome editing was performed to obtain the *ABCA3*-knockout clone as described in the Methods in the main manuscript. For construction of an sgRNA-Cas9 co-expression vector, DNA fragments (Supplementary table. 1) were inserted into pSpCas9(BB)-2A-GFP (px458) vector. MDCK cells were transfected with sgRNA-Cas9 co-expression vector. After cell sorting, each clone was screened based on restriction fragment length polymorphism (RFLP)-based analysis and direct sequencing of the *ABCA3* gene using specific primers (Supplementary table. 1). The obtained *ABCA3*-knockout clone had two mutations in each allele; one was a two-nucleotide deletion at the 5'-terminal of exon 4, which results in a frame-shift mutation, and the other was a one-nucleotide deletion at the splicing site adjacent to exon 4, which causes the deletion of exon 4 in the mRNA. The deletion was confirmed by direct sequencing of cDNA derived from the *ABCA3*-knockout clone using a specific primer (Supplementary table. 1). cDNA with the correct sequence was not detected in the clone.

b, c The effects of *ABCA3* gene knockout on the antiviral activity of PVF-tet (**b**) and the HA accumulation induced by PVF-tet (**c**). Parental MDCK cells or the *ABCA3*-knockout clone were infected with IAV strain PR8 at 10 MOI for 1 h. After washing, the cells were cultured in the presence or absence of PVF-tet (20 μM). After 24 h of infection, cell viability was measured by the cytopathicity assay (**b**). Data are presented as a percentage of the control value without infection (mean ± SEM of three independent experiments). *P<0.05; **P < 0.01 (by two-sided Student's *t*-test). After 16 h of infection, the cell lysates were analyzed by western blotting using specific antibodies to measure the amount of cellular HA and β-actin (**c**).

d The effects of *ABCA3* gene knockout on the acidification (left panel) and average size (right panel) of the HA-containing structure induced by PVF-tet. Parental MDCK cells or the *ABCA3*-knockout clone were infected with IAV strain PR8 at 10 MOI for 1 h. After washing, the cells were cultured in the presence of PVF-tet (20 μM) for 15 h. Acidification of the structure was analyzed using Lysotracker. Fluorescent images were analyzed using laser scanning confocal microscopy (left panel). Insets show magnified fields. The average diameter of the HA-positive and Lysotracker-positive structure was measured using ImageJ software (National Institute of Health). Data are presented as a percentage of the total number of structures (mean ± SD of three independent experiments, using at least 150 cells in each experiment).

Scale bar represents 20 μm. Source data are provided as a Source Data File.

Supplementary Figure 12

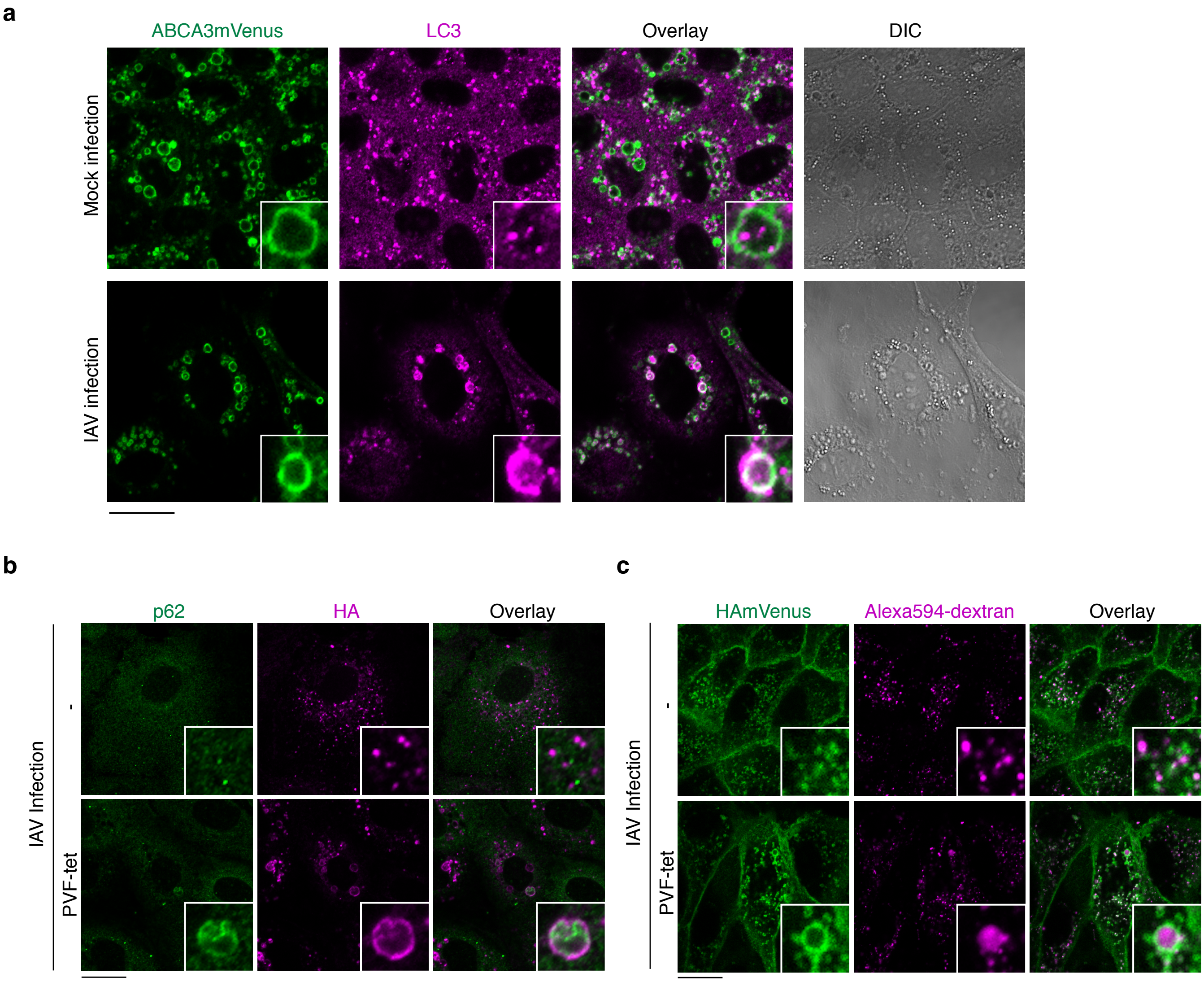

Supplementary Figure 12. Detection of autophagosome/amphisome markers in the inducible amphisomes.

a Co-localization of ABCA3mVenus and LC3. MDCK clone 6 was infected with IAV strain PR8 at 10 MOI or not for 1 h. After washing, the cells were cultured for 15 h. Co-localization of ABCA3mVenus and LC3 was analyzed by immunocytochemical staining. Insets show magnified fields.

b MDCK cells were infected with IAV strain PR8 at 10 MOI for 1 h. After washing, the cells were cultured in the presence or absence of PVF-tet (20 μM) for 15 h. Co-localization of HA and p62 was analyzed by immunocytochemical staining.

c MDCK cells stably expressing HAmVenus were infected with IAV strain PR8 at 10 MOI for 1 h. After washing, the cells were cultured in the presence or absence of 20 μM PVF-tet for 15 h. The cells were treated with Alexa594-dextran (250 μg/ml) for 1 h and then fixed. Co-localization of HAmVenus and Alexa594-dextran was analyzed using laser scanning confocal microscopy.

Scale bars represent 20 μm.

Supplementary Figure 13

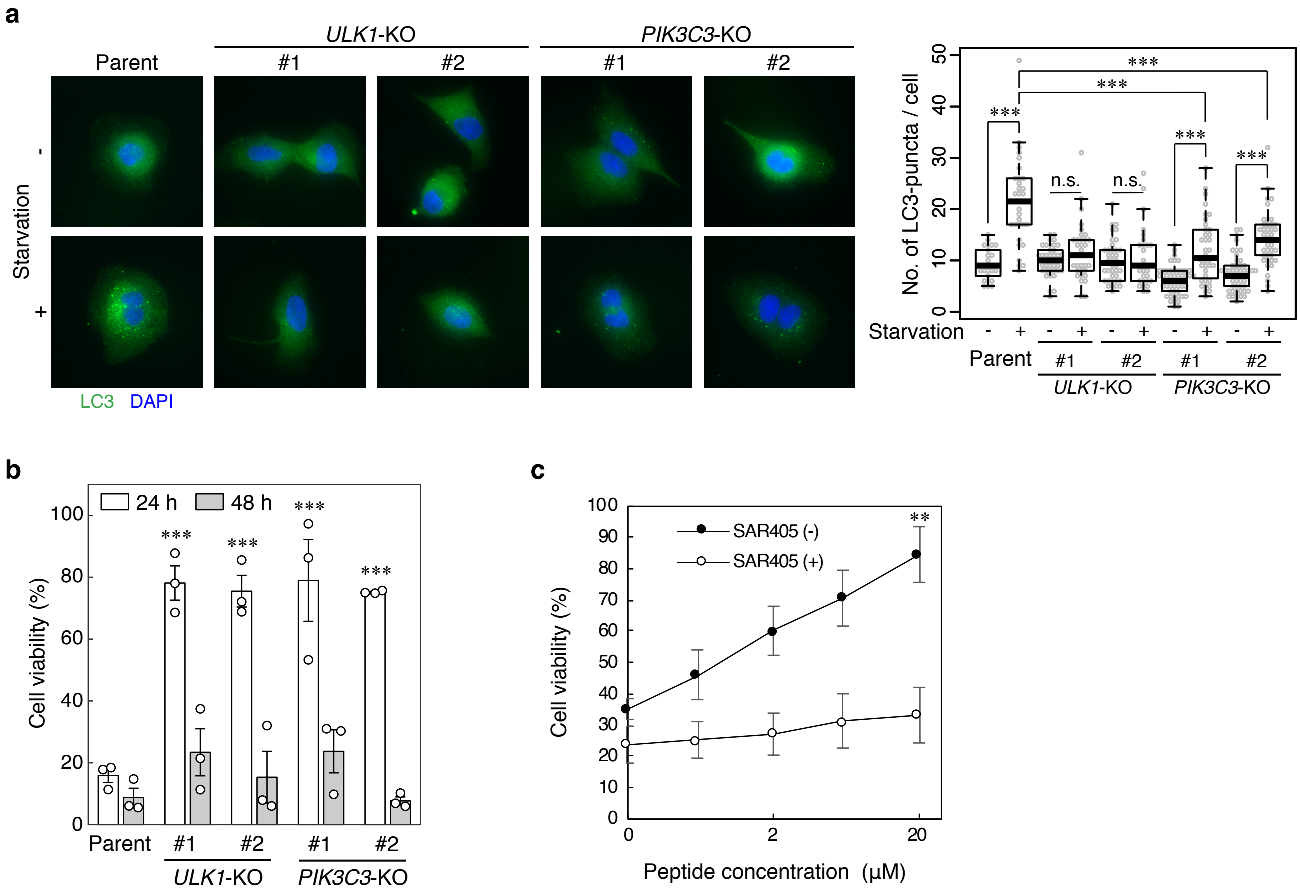

Supplementary Figure 13. Characterization of MDCK-derived *ULK1*- or *PIK3C3*-knockout clones, and the effect of knockouts on the anti-IAV activity induced by PVF-tet.

a The effects of *ULK1* or *PIK3C3* knockout on starvation-induced autophagy. Parental MDCK cells or each knockout clone were cultured in Minimum Essential Medium supplemented with 10% heat-inactivated FBS, 50 units/ml penicillin, and 50 μg/ml streptomycin. After washing, the cells were cultured in the same medium [starvation (-)] or Hank's balanced salt solution containing 0.5 mM Mg^{2+} and 1.3 mM Ca^{2+} [starvation (+)] for 24 h. Localization of LC3 was analyzed by immunocytochemical staining. Fluorescent images were analyzed using an ORCA-ER CCD digital camera (Hamamatsu Photonics) (left panel), and the number of LC3(+)-puncta in each cell was quantified with at least 25 cells (right panel). Boxplot elements are defined as follows: center line, median; box limits, upper and lower quartiles; whiskers, 1.5× interquartile range. Each dot represents the number of LC3(+)-puncta in each cell. ***$P < 0.001$ (by Mann–Whitney U test). The P-values were adjusted by Bonferroni correction. n.s., not significant.

b The effects of *ULK1* or *PIK3C3* knockout on IAV-induced cytopathicity. Parental MDCK cells or each knockout clone was infected with IAV strain PR8 at 10 MOI for 1 h. After washing, cells were cultured for 23 h or 47 h, and then cell viability was measured by the cytopathicity assay. Data are presented as a percentage of the control value without infection (mean ± SEM of three independent experiments). ***$P < 0.001$ (compared with parental cells at 24 h by ANOVA followed by one-sided Dunnett's test). n.s., not significant.

c The effects of SAR405, a specific inhibitor for class III PI3K, on the antiviral activity of PVF-tet were assessed. MDCK cells were infected with IAV strain PR8 at 10 MOI for 1 h. After washing, the cells were treated with or without SAR405 (100 nM) for 23 h in the presence of the indicated amounts of PVF-tet, and then cell viability was measured. Data are presented as a percentage of the control value without infection (mean ± SEM of four independent experiments). **$P < 0.01$ (by two-sided Student's *t*-test).

Source data are provided as a Source Data File.

Supplementary Figure 14

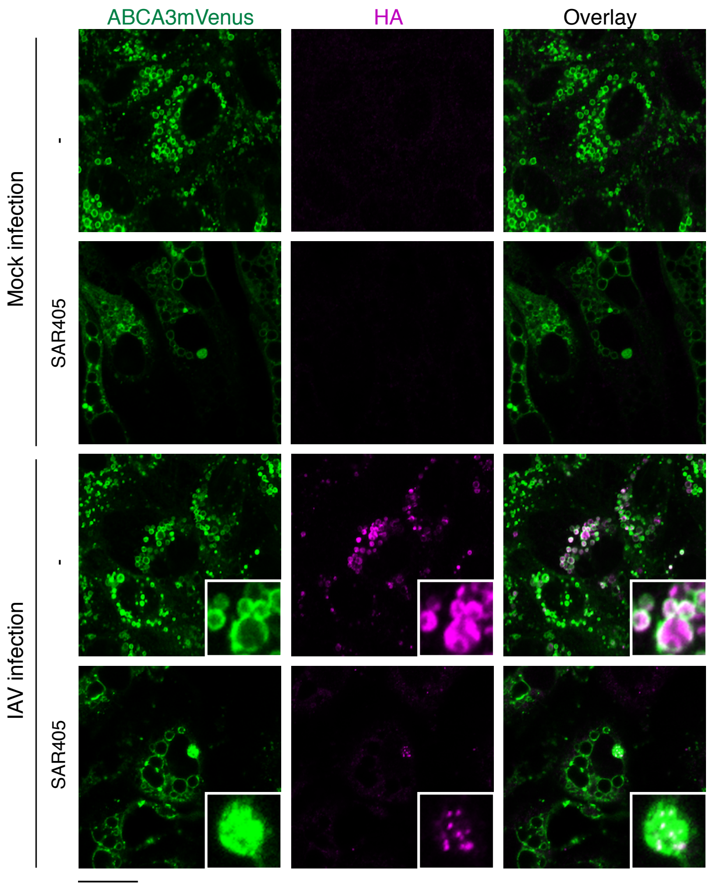

Supplementary Figure 14. SAR405 inhibits the accumulation of HA into the ABCA3-positive structure.

MDCK clone 6 was infected with IAV strain PR8 at 10 MOI or not for 1 h. After washing, the cells were treated with or without SAR405 (100 nM) for 15 h. Co-localization of ABCA3mVenus and HA was analyzed by immunocytochemical staining. Insets show magnified fields. Scale bar represents 20 μm.

Supplementary Figure 15

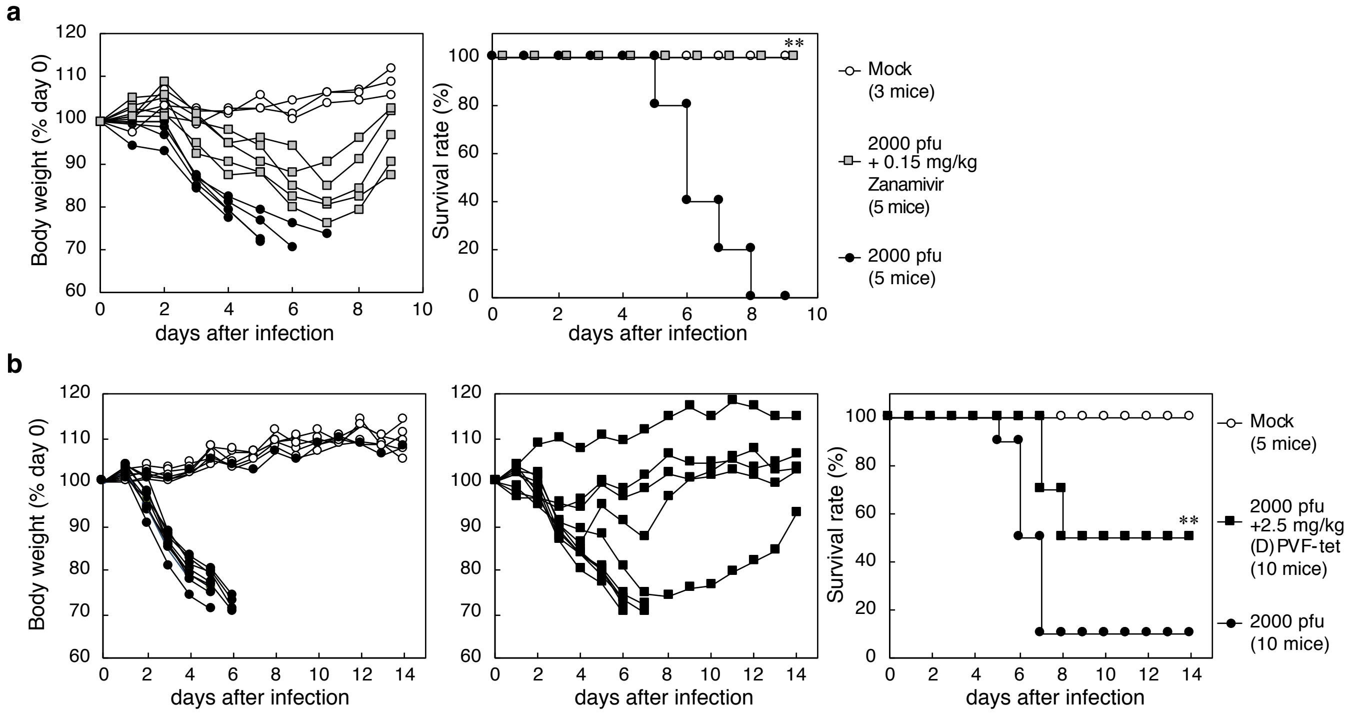

Supplementary Figure 15. (D)PVF-tet rescues mice from the lethality of IAV infection.

a Female BALB/c mice were intranasally infected or not infected (Mock) with 2000 pfu IAV strain PR8 together with or without 0.15 mg/kg zanamivir (Tokyo Chemical Industry Co., Ltd.). The body weight of each mouse is presented as a percentage of the value at day 0 (left panel). The survival rate of each group is shown (right panel). Each group contained 3–5 mice. **P < 0.01 (by Log rank test).

b Female BALB/c mice were intranasally infected or not infected (Mock) with 2000 pfu IAV strain PR8. After 6 h of infection, mice were intranasally treated with 2.5 mg/kg (D)PVF-tet. The body weight of each mouse is presented as a percentage of the value at day 0 (left panel). The survival rate of each group is shown (right panel). Each group contained 5–10 mice. **P < 0.01 (by Log rank test).

Source data are provided as a Source Data File.

Supplementary Figure 16

a

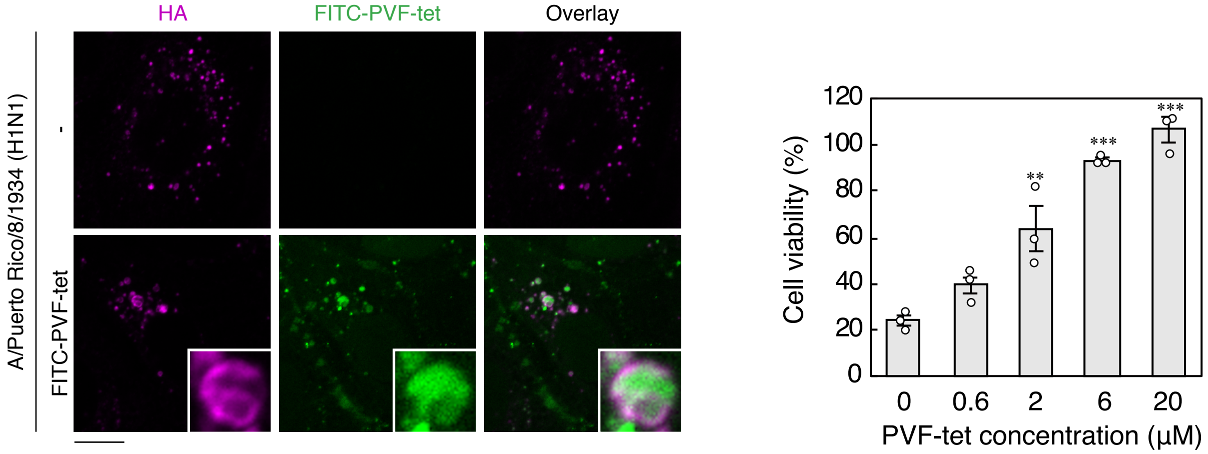

b

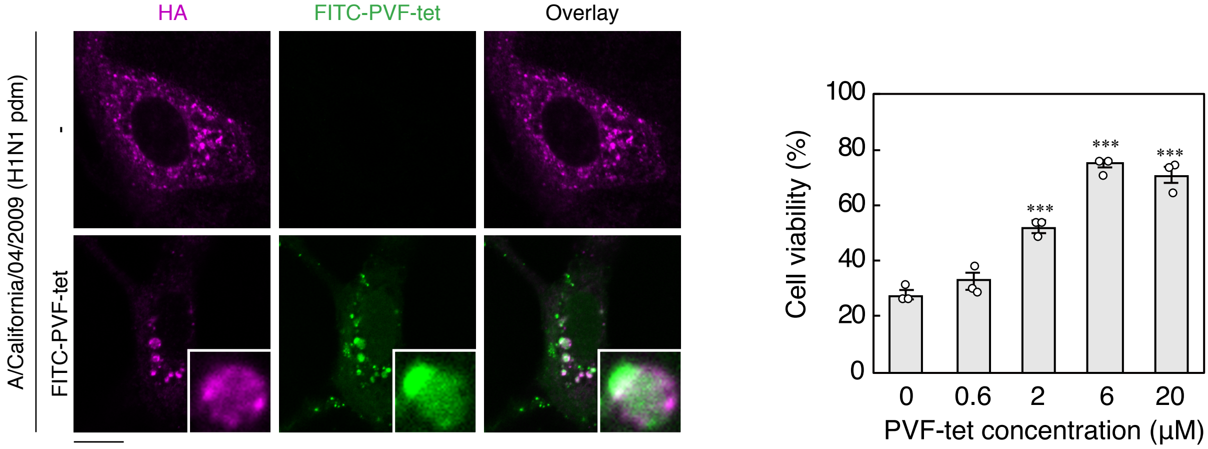

c

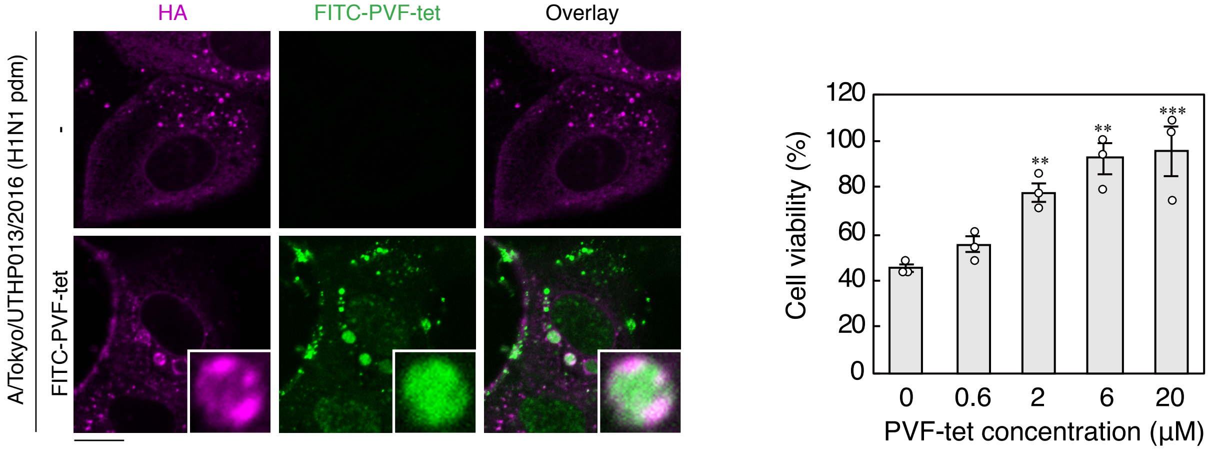

d

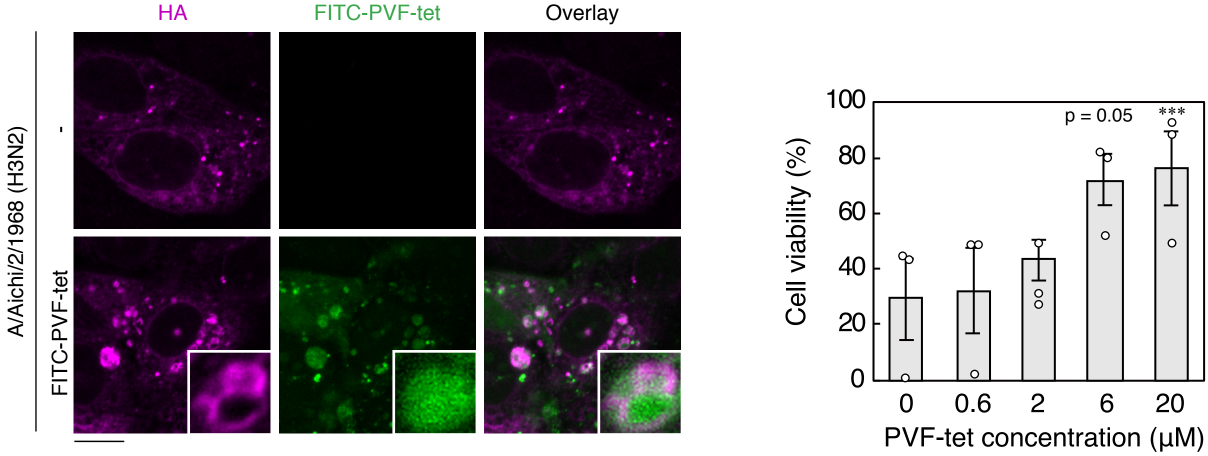

Supplementary Figure 16. PVF-tet reduced the cytopathicity of a broad range of IAV strains through the formation of inducible amphisomes.

a-d (left panels) MDCK cells were infected for 1 h with IAV strains (**a**) PR8 (10 MOI), (**b**) mouse-adapted A/California/04/2009 (H1N1 pdm, 10 MOI), (**c**) A/Tokyo/UTHP013/2016 (H1N1 pdm, 25 MOI), or (**d**) mouse-adapted A/Aichi/2/1968 (H3N2, 4 MOI). The cells were then washed and cultured for 15 h in the presence or absence of FITC-labeled PVF-tet (5 μM). Co-localization of HA and FITC-labeled PVF-tet was analyzed by immunocytochemistry using anti-H1HA antibody (Genetex) or anti-H3HA antibody (Genetex). (right panels) MDCK cells were infected with IAV strains for 1 h as described above. The cells were then washed and cultured for 23 h in the presence of the indicated amounts of PVF-tet. The cell viability was then measured. Data are presented as a percentage of the control value without infection (mean ± SEM of three independent experiments). $^{**}P < 0.01$; $^{***}P < 0.001$ (compared with no PVF-tet treatment by ANOVA followed by one-sided Dunnett's test).

Scale bars represent 20 μm. Source data are provided as a Source Data File.

Supplementary Figure 17

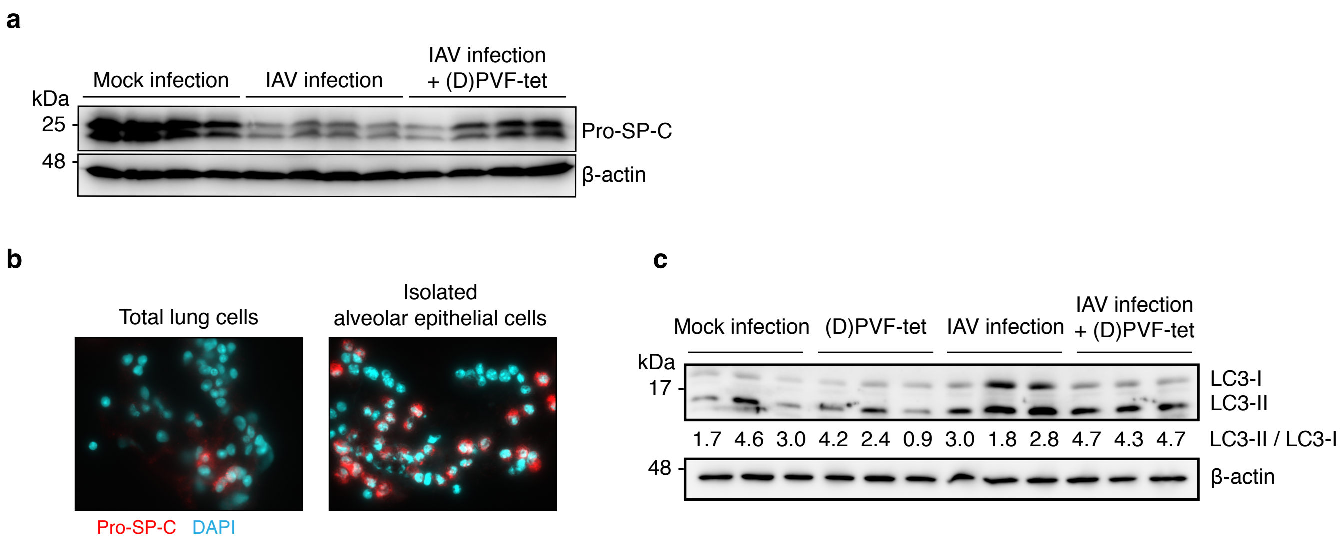

Supplementary Figure 17. (D)PVF-tet rescues mice from the lethality of IAV infection by specifically targeting AT-II cells through autophagic machinery.

a Female BALB/c mice were infected or not infected (Mock) with 2000 pfu IAV strain PR8 with or without 2.5 mg/kg (D)PVF-tet. After 3 days of infection, mice were euthanized. The lungs were collected and then homogenized in 1 ml lysis buffer using Micro Smash MS-100R (TOMY Seiko, Japan) with a zirconia bead (φ5.0 mm). The lysates were centrifuged, and the obtained supernatants were analyzed for the expression of Pro-SP-C by western blot using specific antibodies (Abcam).

b-c Female BALB/c mice were infected or not infected (Mock) with 2000 pfu IAV strain PR8 together with or without 2.5 mg/kg (D)PVF-tet. After 40 h of infection, alveolar epithelial cells were isolated using the immuno-magnetic isolation methods described as follows. Mice were euthanized with an excess amount of isoflurane and perfused with 10 ml PBS via the right ventricle. The lungs were instilled with 3 ml DMEM (Gibco) containing 10% heat-inactivated FBS and 10 mg/ml Dispase II (Godoshusei,. Co., Ltd.) and then 0.5 ml 1% seaplaque agarose, followed by rapid cooling in crushed ice. The lungs were incubated in 0.5 ml DMEM at room temperature for 45 min and then minced. The dissociated cells (Total lung cells) were filtered with a 70-μm Cell Strainer (Falcon), and then labeled with biotinylated anti-CD16/32 and anti-CD45 antibodies (Biolegend) at 4°C for 45 min. After washing, the labeled cells were removed with 25 μl streptavidin-immobilized Dynabeads MyOne T1 (Invitrogen). The remaining cells, mainly consisting of alveolar epithelial cells and pulmonary fibroblasts, were labeled with biotinylated anti-EpCAM antibody (Biolegend) at 4°C for 45 min. After washing, the labeled cells were collected with 25 μl streptavidin-immobilized Dynabeads (Isolated alveolar epithelial cells). Intracellular Pro-SP-C (an alveolar type-II epithelial cell marker) was detected by immunocytochemistry in the total lung cells or in the isolated alveolar epithelial cells (**b**). The isolated alveolar epithelial cells were lysed in lysis buffer. The expression of LC3 or β-actin in the lysates were analyzed by western blot, and the ratio (LC3-II/LC3-I) was quantified using ImageJ software. Each group contained 3 mice. (**c**). Source data are provided as a Source Data File.

Supplementary Figure 18

MA-RRPVNHD-tet (PVD-tet)

Mw: 5444.2

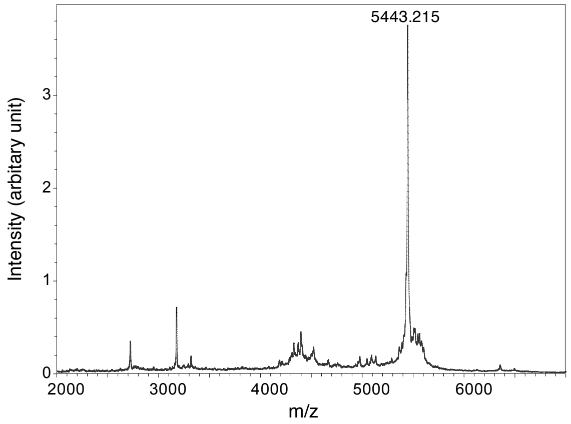

MA-RRPMNHH-tet (PMH-tet)

Mw: 5660.2

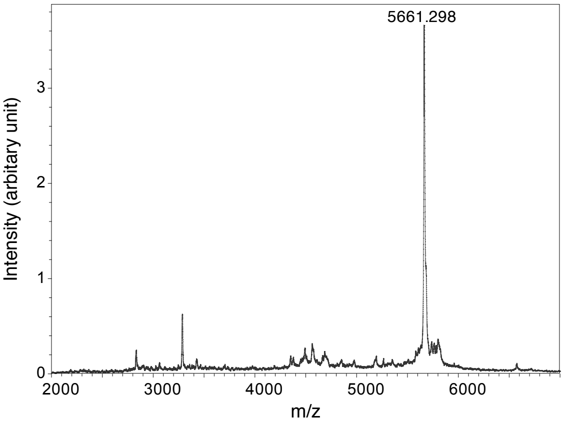

MA-RRPVNHN-tet (PVN-tet)

Mw: 5440.3

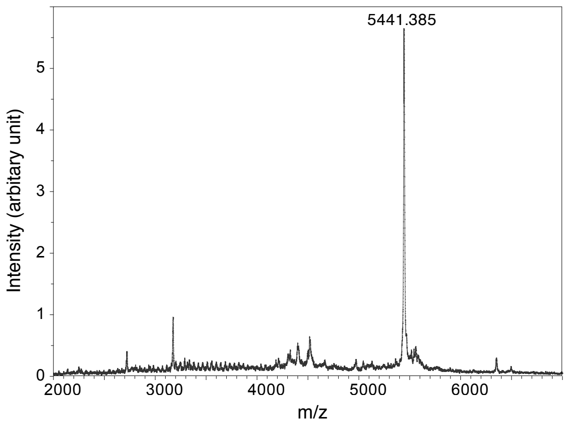

MA-RRPVNHP-tet (PVP-tet)

Mw: 5372.3

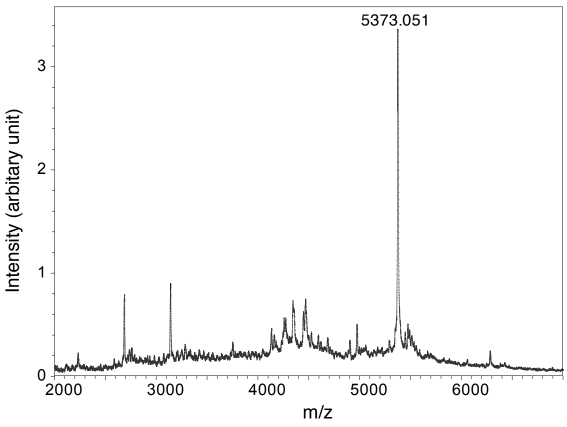

MA-RRPVNHF-A (PVF-monomer)

Mw: 1197.7

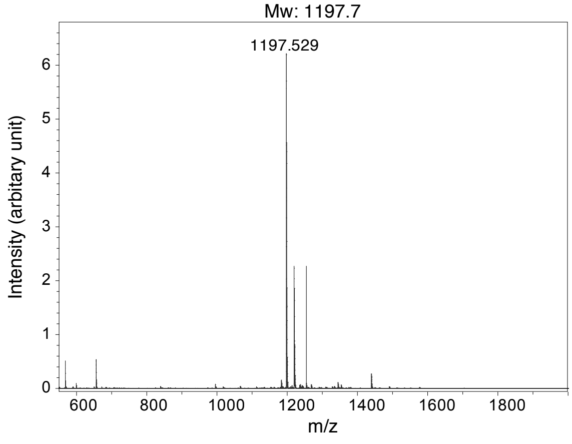

Supplementary Figure 18. Mass spectral characterization of the synthesized peptides.

The synthesized peptides were validated by mass spectrometry analysis using the autoflexII TOF/TOF system (Bruker Daltonics).

Supplementary References

1 Tatsumi R. & Hattori A. Detection of giant myofibrillar proteins and nebulin by electrophoresis in 2% polyacrylamide slab gels strengthened with agarose. *Analytical Biochemistry* **224**, 24-31, doi: 10.1006/abio.1995.1004 (1995)

2 Edward HC. & Ervin F. Purification of influenza virus by haemadsorption and ultracentrifugation. *Protocol Exchange,* doi:10.1038/protex.2014.027 (2014)

3 Sakai T. *et al.* Dual wavelength imaging allows analysis of membrane fusion of influenza virus inside cells. *Journal of Virology* **80**, 2013-2018, doi: 10.1128/JVI.80.4.2013-2018.2006 (2006)

4 Sugiyama K. *et al.* pp32 and APRIL are host cell-derived regulators of influenza virus RNA synthesis from cRNA. *Elife*, pii: 08939, doi:10.7554/eLife.08939 (2015)

5 Schneider B. *et al.* Local dynamics of proteins and DNA evaluated from crystallographic B factors. *Acta crystallographica. Section D, Biological crystallography*, **70**, 2413-2419, doi: 10.1107/S1399004714014631 (2014)

6 Pettersen EF. *et al.* UCSF Chimera--a visualization system for exploratory research and analysis. *Journal of Computational Chemistry*, **13**, 1605-1612, doi: 10.1002/jcc.20084 (2004)
