# Supplementary material for: The inducible amphisome isolates viral hemagglutinin and defends against influenza A virus infection
Source: Nat Commun. 2020 Jan 9;11:162. doi: 10.1038/s41467-019-13974-w (PMC6952414; doi:10.1038/s41467-019-13974-w)
Supplement: Supplementary file 3 — Reporting Summary [file 41467_2019_13974_MOESM3_ESM.pdf]

nature research

Corresponding author(s): Kiyotaka Nishikawa

Last updated by author(s): Nov 24, 2019

Reporting Summary

Nature Research wishes to improve the reproducibility of the work that we publish. This form provides structure for consistency and transparency in reporting. For further information on Nature Research policies, see Authors & Referees and the Editorial Policy Checklist .

Statistics

For all statistical analyses, confirm that the following items are present in the figure legend, table legend, main text, or Methods section.

n/a	Confirmed	
☐	☒	The exact sample size (*n*) for each experimental group/condition, given as a discrete number and unit of measurement
☐	☒	A statement on whether measurements were taken from distinct samples or whether the same sample was measured repeatedly
☐	☒	The statistical test(s) used AND whether they are one- or two-sided<br>*Only common tests should be described solely by name; describe more complex techniques in the Methods section.*
☒	☐	A description of all covariates tested
☐	☒	A description of any assumptions or corrections, such as tests of normality and adjustment for multiple comparisons
☐	☒	A full description of the statistical parameters including central tendency (e.g. means) or other basic estimates (e.g. regression coefficient) AND variation (e.g. standard deviation) or associated estimates of uncertainty (e.g. confidence intervals)
☐	☒	For null hypothesis testing, the test statistic (e.g. *F*, *t*, *r*) with confidence intervals, effect sizes, degrees of freedom and *P* value noted<br>*Give P values as exact values whenever suitable.*
☒	☐	For Bayesian analysis, information on the choice of priors and Markov chain Monte Carlo settings
☒	☐	For hierarchical and complex designs, identification of the appropriate level for tests and full reporting of outcomes
☒	☐	Estimates of effect sizes (e.g. Cohen's *d*, Pearson's *r*), indicating how they were calculated

Our web collection on statistics for biologists contains articles on many of the points above.

Software and code

Policy information about availability of computer code

Data collection	ZEN (ZEISS), AQUALite digital imaging software (Hamamatsu Photonics)
Data analysis	SPSS Statistics (ver. 24.0.0.0), R (ver. 3.5.1), 4peaks (ver. 1.7.2), Illustrator (Adobe)

For manuscripts utilizing custom algorithms or software that are central to the research but not yet described in published literature, software must be made available to editors/reviewers. We strongly encourage code deposition in a community repository (e.g. GitHub). See the Nature Research guidelines for submitting code & software for further information.

Data

Policy information about availability of data

All manuscripts must include a data availability statement. This statement should provide the following information, where applicable:

- Accession codes, unique identifiers, or web links for publicly available datasets
- A list of figures that have associated raw data
- A description of any restrictions on data availability

All data are available upon request to the corresponding author.

Field-specific reporting

Please select the one below that is the best fit for your research. If you are not sure, read the appropriate sections before making your selection.

☒ Life sciences ☐ Behavioural & social sciences ☐ Ecological, evolutionary & environmental sciences

For a reference copy of the document with all sections, see nature.com/documents/nr-reporting-summary-flat.pdf

Life sciences study design

All studies must disclose on these points even when the disclosure is negative.

Sample size	No statistical methods were used to determine the sample size. We repeated each in vitro experiment at least three times and results were reproducibly obtained. For the animal experiments, 5-10 mice were supposed to be minimum and sufficient to identify the effect as indicated by the p-values.
Data exclusions	No data were excluded in this study.
Replication	In vitro experiments were independently performed at least three times and all attempts at replication were successful.
Randomization	Mice were randomly assigned to each group in all animal experiments prior to the beginning of the experiments.
Blinding	No blinding method was performed for all experiments. This approach is considered standard for the field such as biochemistry and cell biology, relevant to this study.

Reporting for specific materials, systems and methods

We require information from authors about some types of materials, experimental systems and methods used in many studies. Here, indicate whether each material, system or method listed is relevant to your study. If you are not sure if a list item applies to your research, read the appropriate section before selecting a response.

Materials & experimental systems

n/a	Involved in the study	
☐	☒	Antibodies
☐	☒	Eukaryotic cell lines
☒	☐	Palaeontology
☐	☒	Animals and other organisms
☒	☐	Human research participants
☒	☐	Clinical data

Methods

n/a	Involved in the study	
☒	☐	ChIP-seq
☒	☐	Flow cytometry
☒	☐	MRI-based neuroimaging

Antibodies

Antibodies used	Antibodies were obtained from the vendors as follows: Mouse monoclonal anti-Hemagglutinin (clone C102, Genetex, Cat# GTX28262), Rabbit polyclonal anti-Hemagglutinin (Genetex, Cat# GTX127357), Mouse monoclonal anti-Neuraminidase (clone GT288, Genetex, Cat# 629696), Rabbit polyclonal anti-Matrix protein 2 (Genetex, Cat# 125951), Mouse monoclonal anti-Nucleoprotein (clone HT103, Kerafast, Cat# EMS010), Mouse monoclonal anti-6xHistidine-tag (clone 9C11, Wako, Cat# 015-23094), Mouse monoclonal anti-GFP (clone B-2, Satna Cruz Biotechnology, Cat# sc-9996), Rabbit polyclonal anti-Fluorescein (Molecular Probes, Cat# A-889), Rabbit polyclonal anti-β-actin (MBL International, Cat# PM-053), Mouse monoclonal anti-ABCA3 (clone 3C9, Biolegend, Cat# 911001), Rabbit polyclonal anti-LAMP1 (Abcam, Cat# ab24170), Rabbit polyclonal anti-EEA1 (Thermo Fisher Scientific, Cat# PA1-063A), Rabbit polyclonal anti-Calnexin (Santa Cruz Biotechnology, Cat# sc-11397), Mouse monoclonal anti-p230 (BD Biosciences, Cat# 611280), Mouse monoclonal anti-LC3 (Nano tools, Cat# 0231-100/LC3-5F10), Rabbit polyclonal anti-LC3 (MBL International, Cat# PM036B), Rabbit polyclonal anti-Pro-surfactant protein C (Abcam, Cat# ab90716), Rabbit polyclonal anti-p62 (MBL International, Cat# PM045), Mouse monoclonal anti-HSP47 (clone M16.10A1, Enzo Lifesciences, Cat# ADI-SPA-470-D), Mouse monoclonal anti-GM130 (clone 35/GM130, BD Biosciences, Cat# 610822), Mouse monoclonal anti-GS28 (clone 1/GS28, BD Biosciences, Cat# 611185), Mouse monoclonal anti-ULK1 (clone F-4, Santa Cruz Biotechnology, Cat# sc-390904), Rabbit monoclonal anti-PI3 Kinase Class III (clone D9A5, Cell Signaling Technology, Cat# 4263S), Goat anti-rabbit IgG, HRP-linked antibody (Cell Signaling Technology, Cat# 7074S), Horse anti-mouse IgG, HRP-linked antibody (Cell Signaling Technology, Cat# 7076S), Alexa Fluor 488 goat anti-mouse IgG (Thermo Fischer Scientific, Cat# A-11001), Alexa Fluor 546 goat anti-rabbit IgG (Thermo Fischer Scientific, Cat# A-11010), Alexa Fluor 488 goat anti-rabbit IgG (Thermo Fischer Scientific, Cat# A-11008), Alexa Fluor 546 goat anti-mouse IgG (Thermo Fischer Scientific, Cat# A-11003).
Validation	All purchased antibodies were well validated by the manufactures in their specific data sheets or other researchers in the previous literatures. Prior to using, we tested the reactivity of the purchased antibodies against the samples alongside the positive control ensured in the manufacture's data sheet.

Eukaryotic cell lines

Policy information about cell lines

Cell line source(s)	Parental MDCK cell (ATCC CCL-34) or Sf21 cell were obtained from ATCC or Clontech (Cat# 631402), respectively. MDCK cell expressing HAmVenus, MDCK cell expressing ABCA3mVenus, ABCA3-knockout MDCK cell, PIK3C3-knockour MDCK cell and ULK1-knockout MDCK cell were generated in this study.

Authentication	All MDCK cell lines used in this study were negative for the contamination of at least human- or mouse-derived cell lines, as tested by previously established procedure (Cytotechnology., Vol. 61., p81-92., (2009)). This is based on the length of PCR-amplified amplicon of cytochrome c gene that varies among species.
Mycoplasma contamination	The cell lines used in this study were negative for Mycoplasma contamination test.
Commonly misidentified lines (See ICLAC register)	No commonly misidentified cell lines were used in this study.

Animals and other organisms

Policy information about studies involving animals; ARRIVE guidelines recommended for reporting animal research

Laboratory animals	Female BALB/cCrSlc 6-8 wk, raised under specific pathogen free condition.
Wild animals	This study did not involve wild animals.
Field-collected samples	This study did not involve samples collected from the field.
Ethics oversight	All animal experiments were approved by the animal ethics committee of Doshisha University prior to their commencement, and performed in accordance with approved protocol.

Note that full information on the approval of the study protocol must also be provided in the manuscript.
